# Response to climate change of montane herbaceous plants in the genus *Rhodiola* predicted by ecological niche modelling

**DOI:** 10.1038/s41598-018-24360-9

**Published:** 2018-04-12

**Authors:** Jianling You, Xiaoping Qin, Sailesh Ranjitkar, Stephen C. Lougheed, Mingcheng Wang, Wen Zhou, Dongxin Ouyang, Yin Zhou, Jianchu Xu, Wenju Zhang, Yuguo Wang, Ji Yang, Zhiping Song

**Affiliations:** 1 The Ministry of Education Key Laboratory for Biodiversity Science and Ecological Engineering, Institute of Biodiversity Science, Institute of Botany, Fudan University, Shanghai, 200438 China; 2 Key Laboratory of Plant Diversity and Biogeography of East Asia, Kunming Institute of Botany, Chinese Academy of Science, Kunming, 650201 China; 3 Department of Biology, Queen’s University, Kingston, Ontario K7L 3N6 Canada

## Abstract

Climate change profoundly influences species distributions. These effects are evident in poleward latitudinal range shifts for many taxa, and upward altitudinal range shifts for alpine species, that resulted from increased annual global temperatures since the Last Glacial Maximum (LGM, ca. 22,000 BP). For the latter, the ultimate consequence of upward shifts may be extinction as species in the highest alpine ecosystems can migrate no further, a phenomenon often characterized as “nowhere to go”. To predict responses to climate change of the alpine plants on the Qinghai-Tibetan Plateau (QTP), we used ecological niche modelling (ENM) to estimate the range shifts of 14 *Rhodiola* species, beginning with the Last Interglacial (ca. 120,000–140,000 BP) through to 2050. Distributions of *Rhodiola* species appear to be shaped by temperature-related variables. The southeastern QTP, and especially the Hengduan Mountains, were the origin and center of distribution for *Rhodiola*, and also served as refugia during the LGM. Under future climate scenario in 2050, *Rhodiola* species might have to migrate upward and northward, but many species would expand their ranges contra the prediction of the “nowhere to go” hypothesis, caused by the appearance of additional potential habitat concomitant with the reduction of permafrost with climate warming.

## Introduction

Climate change has profoundly impacted the distributions of species across the globe^1^. Rising temperatures of the last few decades have shifted the latitudinal and altitudinal ranges of many species^2^. This is particularly concerning for alpine species, for which there may not be sufficient suitable alpine habitats at higher altitudes to facilitate their migration. This phenomenon has been termed the “nowhere to go” hypothesis^3,4^. Chen *et al*.^5^ conducted a meta-analysis of over 1,000 species and found that the median rate of increased altitudinal range shifting was 11 meters per decade, while latitudinal range shifts showed a median rate of 16.9 kilometers poleward per decade (animals only). Lenoir *et al*.^6^ investigated 171 plant species in European montane regions, and found that mean elevation of ranges had moved upward by 29 meters per decade, coincident with rapidly rising temperatures occurring after 1986. These altitudinal shifts were faster for species adapted to montane regions than for more broadly-distributed species and for herbaceous plants compared to woody plants^6,7^. These studies collectively suggest that, under future climate warming scenarios, montane species, especially herbaceous plants, may face the aforementioned “nowhere to go” predicament. However, other researchers predict that global climate warming may drive snowlines upward, exposing new areas of suitable habitat that could facilitate alpine species’ upward migration; thus the notion of “nowhere to go” may be an oversimplification of the future of alpine taxa^8^. For example, Holzinger *et al*.^9^ reported that species richness of vascular plants in montane communities increased by 11% per decade under climatic warming. Clearly more research is required to fully explore the effects of global warming on alpine plants.

**Figure 1:**
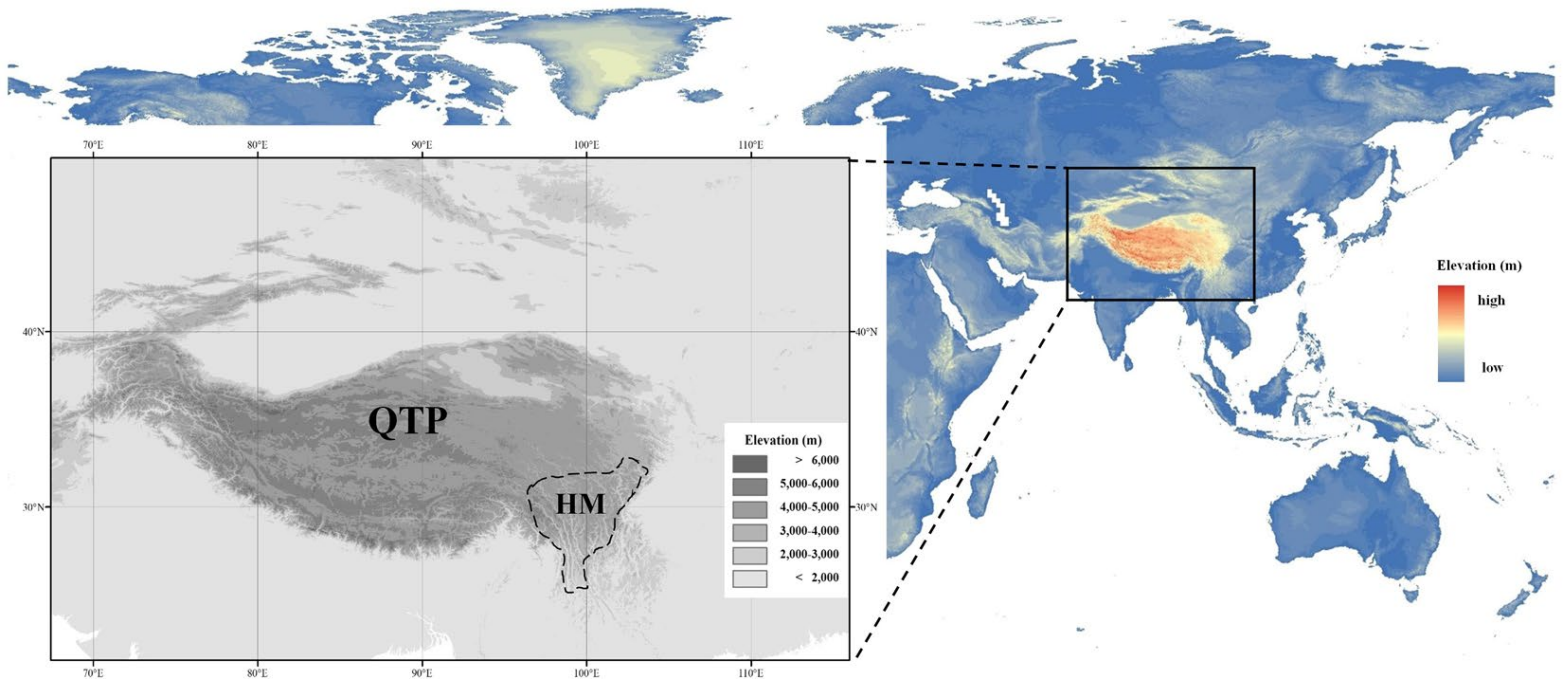
Location and elevation of *Rhodiola* distributions within the Qinghai-Tibetan Plateau (QTP) and the Hengduan Mountains (HM). Elevation data were downloaded from WorldClim Dataset (www.worldclim.org/bioclim). The map was processed by ArcGIS ver 10.2 (ESRI, Redlands, California, USA) (http://www.esri.com/).

The Qinghai-Tibetan Plateau (QTP) is the highest alpine ecosystem in the world, and perhaps one of the most sensitive to climate change. The temperature increase in the QTP has been faster than the mean temperature increases in the Northern Hemisphere as well as in other regions at the same latitude^10,11^. The QTP and environs (like Hengduan Mountains (HM), Fig. 1) are also a key global biodiversity hotspot containing one of the richest alpine flora in the world^12^. Recent phylogeography studies of QTP plants reveal that Quaternary glaciations, especially the Last Glacial Maximum (LGM, ca. 22,000 years before present (BP)), have profoundly influenced the distributions of these species^13,14^. These studies rarely discuss how QTP plants will respond to future climate change, a major focus of our paper.

*Rhodiola* is a perennial herb genus comprising nearly 70 species, which are primarily distributed in the QTP and neighboring mountains^15^. Molecular phylogenetic analyses suggest that *Rhodiola* originated in the QTP about 21.0 Mya and rapidly diversified beginning 12.1 Mya coincident with the uplift of the QTP^15^. The genus subsequently expanded into adjacent regions, with a handful of species (e.g. *R. rosea*) dispersing to other parts of the globe^16^. The evolutionary history and contemporary geographic ranges suggest that *Rhodiola* species are adapted to low temperatures, making them an ideal model for investigating the response of montane herbaceous species to climate change. To date, the genealogical patterns of three *Rhodiola* species (*R. alsia*, *R. dumulosa* and *R. kirilowii*) of the QTP have been investigated^17–19^ and these studies suggested that contemporary *Rhodiola* distributions have been highly influenced by the LGM temperature where they expanded from refugia in the southeastern QTP and HM^17–19^. A limitation of these studies is that they do not utilize ecological niche modelling (ENM) with their molecular data, and thus exhibit only a cursory understanding of how changing environments might have shaped contemporary distributions and phylogeographic patterns.

ENM is based on the concept of the ecological niche, which associates a set of environmental variables to the ability of a species to persist in an environment, and is related to the fitness of a species^20^. These environmental variables (e.g. climate, soil moisture and nutrients, etc.) constrain the distribution and abundance of a species^21,22^. Using occurrence data of a particular species, ENM can be used to project its ecological niche back into palaeoclimatic scenarios, and also into future climate scenarios, predicting potential distributional patterns under the assumptions of niche conservatism^23–25^.

In this study, framing our research questions using a molecular phylogeny of *Rhodiola*^15^, we focus on 14 species with sufficient occurrence records for ENM analysis (Table 1). These species belong to different *Rhodiola* clades that vary in their distributions and breeding systems (Table 1)^15^, allowing us to test for the effects of evolutionary history, ecology niche differentiation, and reproductive strategies on plant responses to climate change. The specific aims of our study are to: 1) test which climatic factors underlie the distributions and range shifts of *Rhodiola* species in different periods (Last inter-glacial (LIG) ca. 120,000–140,000 years BP, LGM ca. 22,000 years BP, current, and future (2050), and 2) test the predictions of the “nowhere to go” hypothesis under future climate change scenarios.

**Table 1:** Ecological information for the 14 *Rhodiola* species^14^ and occurrence data used for ENM.

Species	Habitat	Distribution	Elevation (m)	Breeding system	Clade in genus-level phylogeny	Records
*R. alsia*	*Rhododendron* forests, rocky slopes	Eastern QTP and Hengduan Mountains (G_QTP_)	2450–5300	Dioecism	Clade2	71
*R. bupleuroides*	Thickets, grassy places, rock crevices on slopes	Eastern and southern QTP and Hengduan Mountains (G_QTP_)	2300–5920	Dioecism	Clade2	200
*R. chryanthemifolia*	Grasslands, rocks, rock crevices	Eastern and southern QTP and Hengduan Mountains (G_QTP_)	2500–5140	Monoecism	Clade1	71
*R. crenulata*	Thickets, grassland slopes, schist on mountain slopes, rocky places, rock crevices	Eastern and southern QTP and Hengduan Mountains (G_QTP_)	2700–5850	Dioecism	Clade2	96
*R. dumulosa*	Rocky slopes	From eastern and southern QTP to northeastern China (G_wide_)	1570–5700	Monoecism	Clade2	93
*R. fastigiata*	Rocky slopes	Southern and eastern QTP, Hengduan Mountains (G_QTP_)	2460–5600	Dioecism	Clade2	161
*R. forrestii*	Slopes	Western Sichuan, northwestern Yunnan (G_HM_)	1600–4800	Dioecism	Clade2	41
*R. henryi*	Rocky slopes	Xichuan, Hubei, Gansu, Shanxi, Henan (G_plain_)	500–4200	Dioecism	Clade2	125
*R. himalensis*	Slopes, forests, scrub	Eastern and southern QTP and Hengduan Mountains (G_QTP_)	2600–5300	Dioecism	Clade2	92
*R. kirilowii*	Forest margins, grassy slopes, often in partial shade	Eastern and southern QTP, northeastern China and Xinjiang (G_wide_)	1500–5300	Dioecism	Clade2	174
*R. quadrifida*	Alpine regions, stony slopes, rocks	QTP and the neighboring mountains (G_QTP_)	2500–5200	Dioecism	Clade2	78
*R. sacra*	Grassland slopes, rock crevices on slopes	Southern QTP and Hengduan Mountains (G_QTP_)	2700–5330	Monoecism	Clade1	69
*R. wallichiana*	Forests, rocky slopes	Southern and eastern QTP, Hengduan Mountains (G_wide_)	2500–5100	Monoecism	Clade1	39
*R. yunnanensis*	Forests on slopes	Hengduan Mountains and the neighboring plateau and plain (G_HM_)	1400–4600	Dioecism	Clade2	134

## Results

### Model performance

We used three modelling approaches to predict the distributions of *Rhodiola* species: (1) MEAN ENSEMBLE: using each Global Climate Model (GCM, one GCM for LIG and current, 4 GCMs for LGM, and 17GCMs for 2050, respectively) to do ensemble ENM, and then calculated the mean and standard deviation of all predictions; (2) MMM ENSEMBLE: using the multi-model mean (MMM) of all the GCMs to do ensemble ENM; (3) The maximum entropy algorithm, Maxent: using the MMM of all the GCMs to do Maxent modelling. The Area Under the Receiver Operator Curve (AUC) of ensemble models (MEAN ENSEMBLE and MMM ENSEMBLE) ranged from 0.968 to 0.995 for individual species with our designated groups (clade, distribution and breeding system) (Supplementary Table S1) showing high performance. Maxent also showed good performance in modelling the distributions of *Rhodiola* species: the AUC values ranged from 0.925 to 0.993 and True Skill Statistic (TSS) values from 0.902 to 0.993, both statistics being significantly higher than would be expected at random (all p < 0.0001, Wilcoxon rank sum test) (Supplementary Table S2). Similarly, all training omission (OR) values in Maxent were significantly lower than random predictions (all p < 0.0001, Wilcoxon rank sum test). Comparisons of model AUC values for the genus as a whole (WHOLE, 14 *Rhodiola* species), for each species group, and for constituent species of each species group revealed no significant differences (for all, *p* > 0.05, Wilcoxon rank sum test), indicating that all three modelling approaches performed well irrespective of the grouping.

### Climate variables

The most important bioclimatic variables associated with the distribution of *Rhodiola* species were temperature-related (Isothermality, Mean Temperature of Wettest Quarter, and Mean Temperature of Driest Quarter) (Supplementary Table S3). Isothermality (BIO3) was the most important factor for predicting the distribution of *R. bupleuroides*, *R. crenulata*, *R. fastigiata*, *R. sacra*, G_clade1_ (*Rhodiola* species found primarily in the first major clade in the genus-level phylogeny tree), and G_QTP_ (species mainly confined to the QTP); whereas Mean Temperature of Wettest Quarter (BIO8) was the most important variable for the distributions of *R. alsia*, *R. dumulosa*, *R. himalensi*, *R. kirilowii*, *R. quadrifida* and G_wide_ (species with relatively wide distributions). Mean Temperature of Driest Quarter (BIO9) was the top performing variable for predicting the distributions of *R. chryanthemifolia*, *R. forrestii*, *R. henryi*, *R. wallichiana*, *R. yunanensis*, WHOLE, G_clade2_ (species from the second major clade in the *Rhodiola* phylogeny), G_HM_ (species mainly distributed within the HM), G_plain_ (comprising species not found in the QTP and HM), G_mon_ (comprising monoecious species), and G_dio_ (comprising dioecious species).

The principal components analysis (PCA) of temperature variables generated two temperature axis (Temp1 and Temp2) that explained 55.6% and 32.1% (87.7% cumulatively) of the total variation in the temperature dataset. The PCA of precipitation variables produced two precipitation axis (Prec1 and Prec2) that explained 62.3% and 25.6% (87.8% cumulatively) of total variation in the precipitation dataset. BIO8 (Mean temperature of wettest quarter) and BIO9 (Mean temperature of the driest quarter) both had the largest loadings (contributions to the summed variance) for Temp 1 and were both positive. Mean Diurnal Range (BIO2) and Isothermality (BIO3) showed the highest loadings for Temp2 (Supplementary Table S4). Three precipitation variables, Precipitation of Driest Month (BIO14), Precipitation of Coldest Quarter (BIO19), and Precipitation Seasonality (BIO15) loaded highly on Prec1, with the former two loading positively and the last negatively, whereas both Precipitation of Wettest Month (BIO13) and Precipitation of Warmest Quarter (BIO18) had positive loadings on Prec2 (Supplementary Table S5).

**Figure 2:**
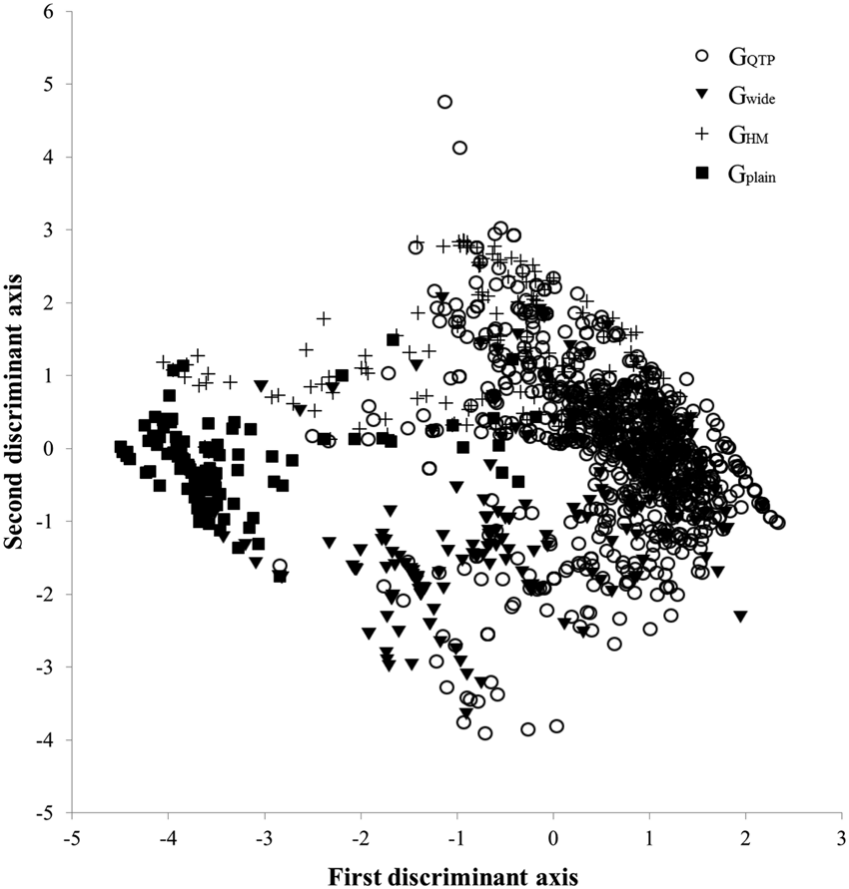
Canonical discriminant analysis (CDA) ordination of the four *Rhodiola* distribution groups: G_QTP_ (○); G_wide_ (▽); G_HM_ (+); G_plain_ (□).

The canonical discriminant analysis (CDA) using Temp1, Temp2, Prec1 and Prec2 produced three discriminant functions, the first explained 76.9% among-group climatic variance, the second 20.1% (Fig. 2). The third explained the remaining 3% of the total variance, suggesting that it could be neglected in subsequent analysis. Of the four variables, Temp2 contributed the most to the first discriminant function, while Temp1 contributed most to the second (Supplementary Table S6).

The Wilks’ Lambda value for each CDA axis was significant at α = 0.05, suggesting that at least some of the 14 focal species occupied geographic ranges that were climatically distinguishable. The first discriminant function best separated G_plain_ and G_QTP_ from other groups (Supplementary Fig. S1). Specifically, G_plain_ had the lowest first discriminant function corresponding to lower Mean Diurnal Range (BIO2) and Isothermality (BIO3), whereas G_QTP_ had the highest first discriminant function relating to highest Mean Diurnal Range (BIO2) and Isothermality (BIO3). For G_plain_ and G_HM_ the second discriminant function distinctly separated these groups from others. G_HM_ individuals tended to score highest on the second discriminant axis, while G_plain_ individuals tended to have intermediate scores, where these two groups had higher values for Mean Temperature of Wettest Quarter (BIO8) and Mean Temperature of Driest Quarter (BIO9).

### Range shifts

**Figure 3:**
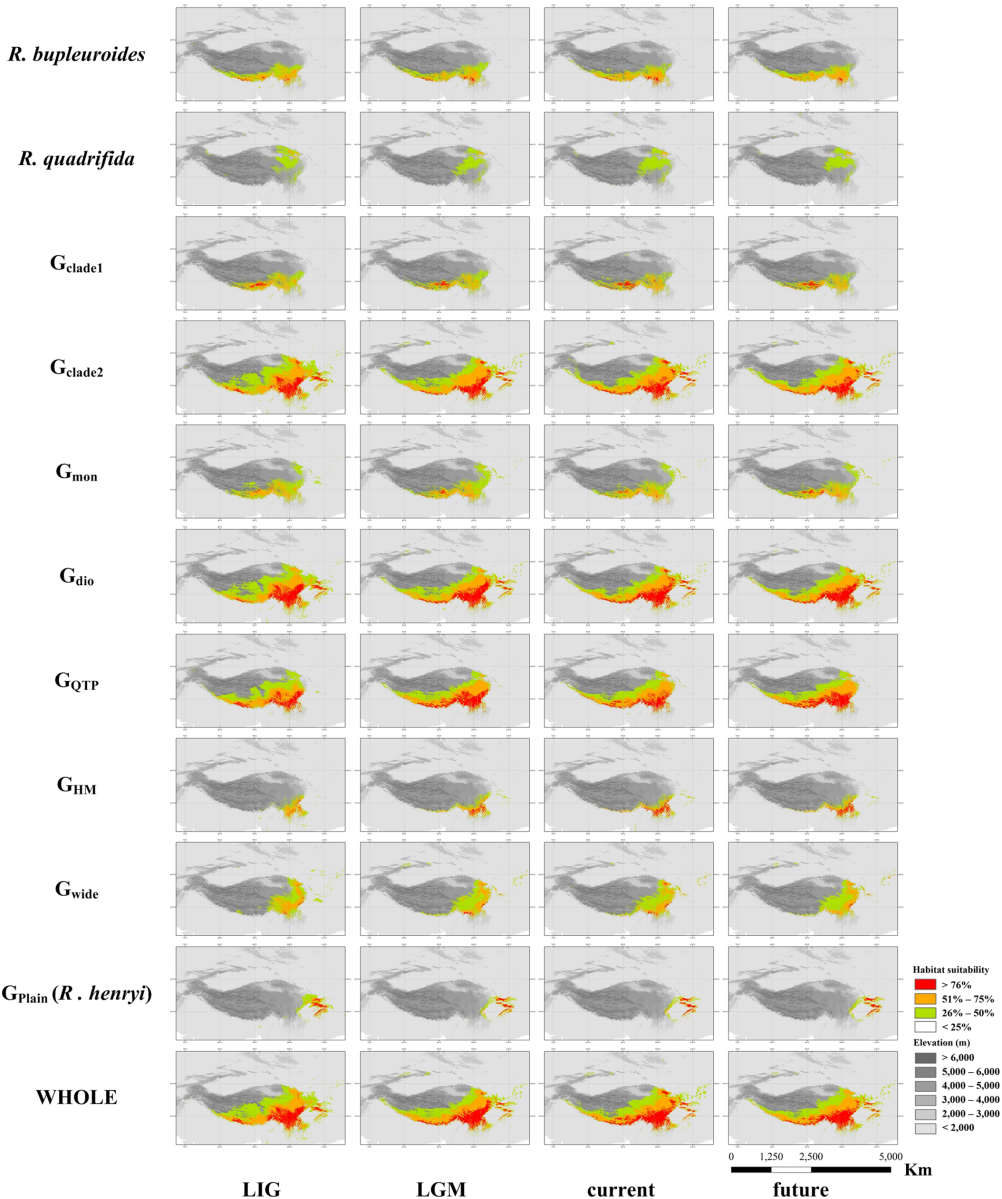
MEAN ENSEMBLE predicted maps for *R. bupleuroides, R. quadrifida*, and species group for the Last inter-glacial (LIG ca. 120,000–140,000 years BP), the Last glacial maximum (LGM ca. 21,000 years BP), current and future (2050). ENM predicted results were processed by ArcGIS ver 10.2 (ESRI, Redlands, California, USA) (http://www.esri.com/), and then integrated using Microsoft Office Visio 2013 (http://office.microsoft.com/visio/).

The predictions generated using MEAN ENSEMBLE, MMM ENSEMBLE, and Maxent all showed that potential distribution of *Rhodiola* varies among species and groups under four climate scenarios: LIG, LGM, current and future (2050 RCP8.5) (Table 2; Fig. 3; Supplementary Figs S2–S4). MEAN ENSEMBLE and MMM ENSEMBLE predicted similar patterns of range shifting (Supplementary Tables S7, S8), which both appear to give more conservative predictions than Maxent. For example, Maxent, on average, predicted relatively larger range shifts and showed some differences in tendency for range shifting to occur (e.g. from current to 2050 for *R. henryi*; see Table 2).

**Table 2:** Predicted suitable area (km^2^) of *Rhodiola* species and species groups for each time period using MEAN ENSEMBLE, MMM ENSEMBLE and Maxent.

	MEAN ENSEMBLE				MMM ENSEMBLE				Maxent			
	LIG	LGM	current	2050	LIG	LGM	current	2050	LIG	LGM	current	future
*R. alsia*	25105	29754	29189	29261	25105	26271	29189	30754	256304	995152	1127870	1126001
*R. bupleuroides*	35105	34687	34381	34554	35105	34239	34381	34873	416941	537477	737503	1151287
*R. chryanthemifolia*	18938	16815	18313	17072	18938	15054	18313	18076	80522	304446	445371	875260
*R. crenulata*	27447	24662	20730	25781	27447	15037	20730	27666	58188	1060539	906215	984226
*R. dumulosa*	12272	9319	9594	8183	12272	7012	9594	10080	164429	392825	323833	606667
*R. fastigiata*	28974	28098	27107	28843	28974	27224	27107	33222	86127	561174	745397	1031277
*R. forrestii*	6068	7336	8082	8113	6068	7891	8082	8556	50141	488778	377406	647803
*R. henryi*	16634	11019	10386	11119	16634	10924	10386	12826	173804	344487	262348	34270
*R. himalensis*	28047	30835	31790	30171	28047	26956	31790	33109	798050	748932	1067350	1270866
*R. kirilowii*	11038	9301	9308	8666	11038	7885	9308	9241	803288	1015632	1212586	1352739
*R. quadrifida*	19668	19659	23196	20056	19668	11754	23196	22277	1920453	2749254	2377665	1800716
*R. sacra*	9116	7972	8152	8260	9116	7548	8152	9260	25017	100974	257310	580758
*R. wallichiana*	5719	5744	7169	6680	5719	5660	7169	7700	765199	1249080	1305554	1642499
*R. yunnanensis*	17391	19182	18540	18587	17391	17754	18540	18777	257468	446269	407214	419536
G_clade1_	31596	28961	27869	27446	31596	28556	27869	28701	351642	308435	554068	1099216
G_clade2_	115702	101676	100741	103019	115702	101137	100741	115453	1234504	1442743	1871221	2032588
G_mon_	43140	42180	36810	38612	43140	39934	36810	40825	1704898	685087	1079352	1813421
G_dio_	110781	96342	95643	96534	110781	96477	95643	113463	1046104	1409270	1758647	1922653
G_QTP_	80186	81275	79792	82687	80186	80406	79792	83951	834374	970820	1434682	1731064
G_HM_	15606	18180	18577	18794	15606	19096	18577	20879	123193	460259	395055	381566
G_wide_	42492	42441	42872	42687	42492	42164	42872	44300	209539	466811	400191	420343
G_plain_	16634	11019	10386	11119	16634	10924	10386	12826	173804	344487	262348	34270
WHOLE	116609	105768	107735	104059	116609	98201	107735	102625	1190909	1372133	1801155	1979687

Between the LIG and LGM, the predictions of MEAN ENSEMBLE and MMM ENSEMBLE indicated no significant range shifts for any species or species group (the values of ΔS1 were near to zero), whereas the results from Maxent showed that most species would have expanded their respective ranges (Table 2; Supplementary Table S9), migrating to lower elevations (Table 3; Supplementary Table S10) and longitudes, but showing increases in the mean latitudes of their distributions (Table 4; Supplementary Tables S11 and S12). Between the LGM and current time points, the combined results of the three modelling approaches showed that most focal *Rhodiola* species expanded their ranges; an exception, *R. henryi*, was predicted to have undergone range contraction over this same period (Fig. 3; Supplementary Table S9; Supplementary Figs S2–S4).

**Table 3:** Predicted mean elevation (m) of *Rhodiola* species and species groups for each time period using MEAN ENSEMBLE, MMM ENSEMBLE and Maxent.

	MEAN ENSEMBLE				MMM ENSEMBLE				Maxent			
	LIG	LGM	current	2050	LIG	LGM	current	2050	LIG	LGM	current	2050
*R. alsia*	4174	4083	4107	4110	4174	4147	4107	4160	3698	3726	4243	4492
*R. bupleuroides*	4155	4158	4160	4203	4155	4136	4160	4181	2893	3222	4158	4500
*R. chryanthemifolia*	3941	3970	3971	3960	3941	3923	3971	3950	3150	2951	3872	4445
*R. crenulata*	4449	4536	4570	4582	4449	4617	4570	4564	4136	4034	4467	4638
*R. dumulosa*	3367	3267	3220	3080	3367	3206	3220	3194	3318	3125	3904	4546
*R. fastigiata*	4086	4053	4069	4093	4086	4063	4069	4140	3603	3540	4175	4570
*R. forrestii*	3155	3176	3176	3199	3155	3169	3176	3296	2638	2438	3310	4222
*R. henryi*	1494	1727	1737	1712	1494	1703	1737	1614	1049	1555	1902	3557
*R. himalensis*	4274	4193	4208	4209	4274	4205	4208	4229	4131	3885	4382	4611
*R. kirilowii*	3311	3201	3188	3146	3311	3100	3188	3111	4192	2714	3818	4311
*R. quadrifida*	4106	4200	4201	4119	4106	4181	4201	4164	4002	4166	4555	4757
*R. sacra*	4403	4310	4310	4320	4403	4309	4310	4319	3090	3734	4142	4561
*R. wallichiana*	4092	4119	4089	4088	4092	4133	4089	4051	2685	2962	4135	4357
*R. yunnanensis*	3109	3097	3099	3146	3109	3131	3099	3107	2571	2324	3076	3994
G_clade1_	4110	4104	4063	4096	4110	4078	4063	4129	2766	3159	4063	4469
G_clade2_	3644	3760	3764	3766	3644	3768	3764	3798	3661	2819	3855	4364
G_mon_	3991	3882	3926	3882	3991	3905	3926	3867	3955	2733	3927	4478
G_dio_	3692	3795	3788	3790	3692	3779	3788	3528	3460	2796	3883	4376
G_QTP_	4249	4282	4237	4290	4249	4268	4237	4259	4101	3645	4291	4527
G_HM_	3010	3063	3061	3107	3010	3054	3061	3129	2622	2357	3106	4016
G_wide_	3702	3762	3765	3749	3702	3779	3765	3785	4359	2702	3836	4343
G_plain_	1494	1727	1737	1712	1494	1703	1737	1614	1049	1555	1902	3557
WHOLE	3723	3794	3780	3797	3723	3757	3780	3685	3698	2833	3885	4366

**Table 4:** Mean longitude/latitude (degree) of *Rhodiola* species and species groups for each time period using MEAN ENSEMBLE, MMM ENSEMBLE and Maxent.

	MEAN ENSEMBLE				MMM ENSEMBLE				Maxent			
	LIG	LGM	current	2050	LIG	LGM	current	2050	LIG	LGM	current	2050
*R. alsia*	98.5/30.5	98.7/31.1	98.7/31.1	98.6/30.9	98.5/30.5	98.7/31.0	98.7/31.1	98.3/31.1	99.3/28.9	96.1/31.1	95.7/31.2	95.0/31.7
*R. bupleuroides*	93.8/28.9	93.7/29.0	93.2/28.9	93.4/28.8	93.8/28.9	93.7/29.1	93.2/28.9	93.2/28.8	95.7/31.9	95.3/28.6	93.5/29.6	92.9/30.6
*R. chryanthemifolia*	96.0/28.6	95.6/28.6	95.7/28.5	95.9/28.5	96.0/28.6	95.5/28.5	95.7/28.5	94.8/28.5	100.3/28.1	97.6/27.9	95.5/29.1	93.7/30.2
*R. crenulata*	94.5/29.5	93.5/29.4	93.4/29.2	93.4/29.3	94.5/29.5	92.0/29.1	93.4/29.2	93.5/29.5	99.4/28.0	93.9/29.7	93.2/30.0	93.1/30.5
*R. dumulosa*	101.2/33.7	101.2/34.6	101.3/34.5	102.1/35.0	101.2/33.7	101.7/34.4	101.3/34.5	101.4/34.5	98.9/31.4	100.4/31.9	96.6/32.9	92.6/33.1
*R. fastigiata*	96.7/29.2	97.1/29.4	96.5/29.2	96.4/29.2	96.7/29.2	96.2/29.3	96.5/29.2	95.2/29.2	100.3/27.9	98.1/29.2	95.5/29.9	93.6/30.7
*R. forrestii*	100.5/27.4	99.8/27.5	99.2/27.4	99.5/27.5	100.5/27.4	99.1/27.5	99.2/27.4	99.1/27.7	97.9/27.3	98.0/27.2	97.2/28.5	95.7/29.9
*R. henryi*	106.6/31.6	106.9/31.3	106.7/31.3	106.9/31.3	106.6/31.6	106.7/31.9	106.7/31.3	106.7/31.8	108.6/28.5	105.7/29.9	105.3/31.9	84.1/35.8
*R. himalensis*	96.0/30.4	97.2/30.7	96.8/30.6	96.9/30.5	96.0/30.4	97.2/30.5	96.8/30.6	96.6/30.5	95.9/29.8	97.1/30.3	94.8/30.8	93.5/31.4
*R. kirilowii*	100.9/34.1	101.9/34.4	101.6/34.6	101.9/34.6	100.9/34.1	102.5/34.6	101.6/34.6	101.7/34.7	95.5/30.4	102.3/32.7	97.2/32.4	94.1/33.2
*R. quadrifida*	98.5/34.4	98.6/33.6	98.3/33.7	98.6/34.3	98.5/34.4	99.2/33.6	98.3/33.7	98.2/34.1	93.0/33.3	90.3/34.4	90.5/33.4	90.3/33.4
*R. sacra*	91.3/28.9	91.6/29.0	91.7/28.9	91.5/28.9	91.3/28.9	91.4/28.9	91.7/28.9	92.0/28.8	101.6/29.7	94.4/29.3	93.3/29.1	92.4/29.8
*R. wallichiana*	90.1/28.5	91.4/28.4	93.2/28.3	93.2/28.3	90.1/28.5	90.3/28.5	93.2/28.3	93.9/28.4	95.1/32.5	90.0/32.0	91.3/30.9	91.1/31.9
*R. yunnanensis*	99.9/28.1	99.5/28.3	99.5/28.1	99.3/28.1	99.9/28.1	99.2/28.2	99.5/28.1	98.9/28.0	99.3/27.2	100.9/28.0	99.5/28.7	97.0/29.7
G_clade1_	95.0/29.1	95.0/29.1	94.3/28.9	94.6/28.8	95.0/29.1	94.3/29.0	94.3/28.9	94.4/28.8	95.8/32.6	97.0/28.4	94.3/29.5	93.0/30.8
G_clade2_	96.6/31.2	95.9/31.0	96.0/31.1	96.0/31.1	96.6/31.2	95.8/31.0	96.0/31.1	95.5/31.2	96.8/29.6	100.6/30.8	95.9/31.6	93.1/32.5
G_mon_	95.7/29.9	96.7/30.2	95.9/29.9	96.0/29.9	95.7/29.9	95.8/30.1	95.9/29.9	95.8/30.0	95.5/31.4	101.8/31.6	96.4/31.1	93.1/32.3
G_dio_	96.4/31.0	95.8/30.8	96.1/30.9	96.1/30.9	96.4/31.0	95.8/30.8	96.1/30.9	96.4/31.5	97.7/29.3	100.5/30.5	95.6/31.3	93.5/32.1
G_QTP_	93.7/30.8	93.5/30.7	94.1/30.8	93.7/30.8	93.7/30.8	93.4/30.7	94.1/30.8	93.7/30.7	96.2/29.9	97.1/30.1	93.7/30.9	93.1/31.8
G_HM_	99.8/27.8	99.7/28.3	100.5/28.1	99.6/28.2	99.8/27.8	100.1/28.2	100.5/28.1	98.8/28.2	98.8/27.2	100.2/27.9	99.5/28.6	97.1/29.7
G_wide_	99.7/31.8	99.1/31.7	99.2/31.5	99.4/31.7	99.7/31.8	99.1/31.1	99.2/31.5	98.8/31.0	93.7/30.9	104.0/33.2	98.0/32.5	94.1/33.3
G_plain_	106.6/31.6	106.9/31.3	106.7/31.3	106.9/31.3	106.6/31.6	106.7/31.9	106.7/31.3	106.7/31.8	108.6/28.5	105.7/29.9	105.3/31.9	84.1/35.8
WHOLE	96.2/31.1	95.6/31.1	96.1/31.4	95.9/31.1	96.2/31.1	95.9/31.1	96.1/31.4	95.9/31.1	96.6/29.7	100.6/30.8	95.8/31.5	93.5/32.3

Between the current to future scenarios, a total of 8, 11 and 10 *Rhodiola* species are predicted to expand their ranges in MEAN ENSEMBLE, MMM ENSEMBLE, and Maxent, with the remaining species predicted to exhibit range contractions; this was especially evident for *R. quadrifida* for which all three modelling approaches predicted range contraction. In addition, we found that ranges were projected to shift more extensively (two or three orders of magnitude) between the current and future scenarios compared to between the LGM to current time points (Supplementary Tables S9–S12). This implies that accelerated climate warming will have marked impacts on future species’ distributions.

Extrapolating from our findings for the future climate warming scenario, the mean upward rate of elevational shifting for *Rhodiola* species would be ca. 0.273 (±0.217) m/year, 0.495 (±0.521) m/year and 7.07 (±5.42) m/year estimated using MEAN ENSEMBLE, MMM ENSEMBLE and Maxent, respectively. The three modelling approaches predicted that the mean northward rate of range movement should be ca. 0.288 (±0.288) km/year, 0.384 (±0.192) km/year and 1.24 (±1.19) km/year for (estimated by 1 degree equals 111 × cos30 km, at about 30° latitude). Maxent had relatively higher predictions than the other approaches. In contrast, some species displayed southward movement, including *R. yunnanensis*, *R. alsia*, *R. himalensis* (Supplementary Table S12).

From the LGM to current and future scenarios, the differences in mean range shifting between sister species versus between species groups were significant for predictions from MEAN ENSEMBLE, MMM ENSEMBLE (except for G_QTP_ and G_wide_), and Maxent (except for G_QTP_ and G_wide_, and G_clade1_ and G_clade2_) (χ^2^ tests, Supplementary Table S13).

## Discussion

### The reliability of model projections

Assertions of historical or future range shifts of species largely depend on the reliability of climatic data used and the ENM modelling process. In order to consider the range of plausible climates and of possible predictions by different models^26^, we used the multi-model mean of all the GCMs to do ENM (MMM ENSEMBLE), and also applied each GCM to do ENM (MEAN ENSEMBLE), and then calculated the mean and standard deviations for all predictions. The predictions of MEAN ENSEMBLE for the LGM and 2050 scenarios overlapped those of MMM ENSEMBLE, implying that modelling with the MMM of the GCMs was reasonable^27,28^. We also found that Maxent^29,30^ models for *Rhodiola* outperformed other sub-model algorithms and predictions generally agreed with those from ensemble models, although Maxent consistently predicted more significant range shifts under future climate warming scenarios. Oppel *et al*.^31^ also reported that Maxent predicted larger areas than ensemble models, the cause of the difference between Maxent and ensemble models calls for further studies.

Our ENM results for three *Rhodiola* species for which there are molecular phylogeographic information (*R. kirilowii*, *R. dumulosa* and *R. alsia*) were consistent with published studies, suggesting that the southeastern QTP and HM were the center from which *Rhodiola* lineages spread to occupy broader geographic ranges from the LGM to the present day (Fig. 3; Supplementary Figs S2–S4)^17–19^. Consistency of these published results with our own findings, increases confidence in our ENM interpretations.

### Upward and northward movement

From the LIG to the LGM, concomitant with climate cooling, most *Rhodiola* species expanded their geographic ranges, consistent with what happened with *R. integrifolia* in North America^32,33^. A similar pattern was also detected in other QTP plants including *Taxus wallichiana* and *Picea likiangensis*^34,35^. Thus, as would be expected of cold-adapted species responding to climate warming^5,6,36^, we found that *Rhodiola* species have generally shifted upward in altitude and also northward since the LGM (Fig. 3; Supplementary Tables S10 and S12; Supplementary Figs S2–S4). Although our estimates of upward rate of movement varied somewhat using different modelling approaches, the mean elevation upward movement of *Rhodiola* (0.273~7.07 m/year) is similar to estimates of upward movement rate for the tree line in the Baima Mountains of the HM (0.84 m/year, between 1923 to 2003)^37^ and montane herbaceous plants in Europe (8 m/year estimated from Fig. 4 in Lenoir *et al*.^6^). We have no estimates for rate of latitudinal movement in plants, although the northward rate of range shifting of *Rhodiola* (0.288~1.24 km/year) is comparable to that of animals (1.69 km/year)^5^.

### Not “nowhere to go”

Contrary to the expectations under the “nowhere to go” hypothesis^3,4^, most *Rhodiola* species broadened their geographic ranges rather than contracting with future climate warming (Fig. 3; Supplementary Table S9; Supplementary Figs S2–S4). DeChaine *et al*.^32^ and Forester *et al*.^33^ found that *R. integrifolia* exhibited a similar pattern of range expansion in North America. Other QTP plants, such as *Hippophae gyantsensis*, *H. rhamnoides* ssp. *yunnanensis*, *H*. *neurocarpa*, *Eriophyton wallichii*, *Thalictrum squamiferum*, *Paraquilegia microphylla* and *Allium przewalskianum* also tend towards range expansion with climate warming^38–40^. Such patterns may be explained by a number of factors. First, these species have relatively small contemporary geographical ranges. For instance, *Rhodiola* is mainly distributed in the eastern and southern regions of the QTP while the more extensive areas available above 4000 m above sea level (asl) in the northern and western parts of the QTP remain uncolonized. Second, warmer temperatures and higher precipitation under future warming conditions could transform unsuitable regions, such as those currently with permafrost, into suitable habitats for *Rhodiola* at higher latitudes and elevations. Moreover, increased vegetation coverage could also play a role in expansion of *Rhodiola* species ranges. Cannone *et al*.^41^ reported that vegetation coverage, particularly that of shrubs, increased dramatically in the alpine belt of Italian Central Alps from 1952 to 2003, coincident with higher precipitation and diminution of permafrost. The extent of shrub coverage has also increased in the Arctic over the past 50 years^42^. *Rhodiola* species typically co-occur with shrubs^15^, and thus we predict that areas suitable for colonization by *Rhodiola* will be increasingly available as shrub vegetation expands in the QTP and environs. Overall then, our results provide evidence that vascular plant species richness in montane communities will increase with climate warming^9^.

In contrast to the patterns evident for its congeners, all predictions generated from MEAN ENSEMBLE, MMM ENSEMBLE and Maxent showed that *R. quadrifida* would have a more restricted geographic range under future climate warming (Fig. 3; Supplementary Table S9; Supplementary Figs S2–S4). This implies that, for higher elevation montane species like *R. quadrifida*, there may be insufficient suitable alpine habitat to facilitate future migration. Thus, for this species at least, projections of future distributions in areas above 4000 m asl on the QTP are consistent with predictions of the “nowhere to go” hypothesis^3,4^.

### Factors underlying species’ distribution

Species’ distributions are shaped by both abiotic (e.g. climatic and edaphic) and biotic factors (e.g. evolutionary history and interspecific competition)^43,44^. ENM is tacitly based on the assumption of niche conservation and is often used to estimate the influence of climate and other abiotic factors on species distributions; however, it can also be used to predict the effects of evolutionary history on species ranges (i.e. examining the evolutionary niche *sensu* Mao & Wang^45^ and Aguirre-Gutiérrez *et al*.^46^). Sister species and closely related species existing in sympatry should have distinct niches for them to coexist, and thus are expected to respond differently to climate change^45,46^. In our study we did detect significantly different patterns of range shifting between sister species and related species within particular clades (Supplementary Table S13). This suggests that there is an effect of evolutionary history, consistent with the view that species diversity of *Rhodiola* resulted from rapid radiation into different environments after their origination in the QTP^16^. In addition, this assertion is consonant with our CDA results that indicated differences in ecological niches among *Rhodiola* species (Fig. 2; Supplementary Fig. S1).

Our ENM showed that, in comparison to precipitation, temperature-related variables are more important influencing factors to *Rhodiola* species distributions, implying that cold-adapted plants exhibit sensitivity to climate warming. Results from our PCA and CAD analyses suggested that divergence in ecological niches accounted for the different distribution patterns among *Rhodiola* species (Fig. 2). Niche breadths of G_plain_ species were distinct from high elevation plants with the former showing broader distributions than the latter. Generally low elevation *Rhodiola* taxa need relatively stable higher temperatures and more homogeneous precipitation throughout the year, whereas higher elevation species are adapted to relatively low temperature, larger diel and seasonal temperature differences and more heterogeneous, and sporadic precipitation. Among the high elevation taxa, the ranges of those within G_HM_ had higher temperatures and summer precipitation levels than taxa within G_QTP_ and G_wide_, with temperature appearing to be more important. The geographic ranges for species within our G_wide_ group exhibited higher temperature stability than those in G_QTP_ (Fig. 2).

Model performance was higher for groups than for the genus in its entirety (WHOLE) (Supplementary Tables S1 and S2), implying that classifications based on distribution patterns may yield more insights about habitat suitability and climate requirements. It was especially notable that the responses to climate change varied between lower versus higher elevation species. For instance, the suitable area available for low elevation *Rhodiola* species (G_plain_) increased from the LIG to the LGM, and then decreased from the LGM to the current and future scenarios. This was in contrast to higher elevation taxa (e.g. G_QTP_) which were predicted to continue to increase from the LIG using Maxent (Supplementary Table S9; Supplementary Fig. S4). These findings together imply that even closely-related species might diverge in their responses to climate change because of niche differentiation^45,46^.

Theoretically, if not self-incompatible, hermaphroditic plants can produce seeds by selfing, restricting pollen mediated gene flow. In contrast, dioecious plants are obligatorily outbreeding and thus have a relatively wider range of pollen mediated gene flow patterns^47,48^. Such differences might explain the range of responses to environmental change^47,48^. MEAN and MMM ENEMBLE predicted that the *Rhodiola* species with divergent breeding systems exhibited differential responses to climate change consistent with the above expectation. However, the results of Maxent implied that the *Rhodiola* species with different breeding systems or no close evolutionary affinities respond similarly to climate change^40^. Species from a biological community will necessarily share adaptations to similar climatic conditions and thus by definition have similar climatic niche requirements^45,46^. Therefore, our study shows that ENM based on communities or functional groups may better reflect the impact of climate on constituent plant taxa within particular regions^49^.

### Conclusion

Our study revealed that *Rhodiola* species were primarily confined to the HM during the LIG, and then expanded into the southeastern QTP during LGM. From the LGM to the present day our results thus indicate that most species expanded to inhabit much broader geographic ranges on the QTP and adjacent regions concomitant with climate warming. These results together imply that high elevation regions in the southeastern QTP and HM were the center of origin and radiation for *Rhodiola*, and the location of refugia. Our analyses suggested that many *Rhodiola* species will not show diminished range sizes under future climate warning as would be predicted by the “nowhere to go” scenario hypothesis. This in part could be because diminution of permafrost at higher altitudes could create large swaths of new, suitable habitat. However, distributions of *Rhodiola* species were projected to show both latitudinal and elevational shifts. Finally, our analyses indicated that species with similar ecological niche requirements will respond similarly to climate change.

## Methods

### Species sampling

We selected *Rhodiola* species for ENM based on the following criteria: (1) phylogeographic data availability, (2) variation in breeding system (dioecious or monoecious), (3) distinction in evolutionary history (sister group vs. different clades in phylogeny tree), (4) dissimilarity in spatial distribution patterns (dispersed or clumped over the QTP), and (5) availability of at least 39 occurrence records. Based on these criteria, a total of 14 species were selected for ENM analysis (Table 1; Supplementary Fig. S5). These species derived from the two main clades at the genus-level phylogeny (Supplementary Fig. S6)^16^ (G_clade1_ comprised three species, G_clade2_ comprised 11 species). Among these 14 targeted species, we included three pairs of sister species (*R. alsia* and *R. fastigiata*, *R. forrestii* and *R. yunnanensis*, *R. henryi* and *R. quadrifida*) that, despite overlapping ranges, show some allopatry^15^. The distributions of the 14 species can be classified into four groups: 1) G_QTP_ = species for which the bulk of their distributions occur on the QTP (*R. alsia*, *R. bupleuroides*, *R. chryanthemifolia*, *R. crenulata*, *R. fastigiata*, *R. himalensis*, *R. quadrifida*, *R. sacra* and *R. wallichiana)*, 2) G_HM_ = species that are mainly distributed within the HM (*R. forrestii, R. yunnanensis)*, 3) G_wide = _species widely distributed from the QTP to northern China (*R. dumulosa* and *R. kirilowii)*, and 4) G_plain_, = not distributed on the QTP or other high elevation (<3000 m) regions (*R. henryi)* (see Table 1; Supplementary Fig. S6). These species can also be classified based on breeding system: dioecy (G_dio_, with 10 species) and monoecy (G_mon_, with 4 species) (Table 1; Supplementary Fig. S6).

Occurrence records were derived from our field expeditions, on-line herbarium databases (Global Biodiversity Information Facility Data Portal, GBIF: www.gbif.org; Chinese Virtual Herbarium Data Portal, CVH: www.cvh.org.cn) and published research (see Supplementary Material1). We removed duplicate records and records with obvious errors in their geographic coordinates and thinned records that occurred within the same 1 km pixel so that only one occurrence remained. After pruning and verifying our data, we were left with 1,444 records for ENM spanning 39 to 200 unique locations (Table 1; Supplementary Fig. S6; see Supplementary Material1).

### Climate variables

We obtained bioclimatic data to test our predictions from the WorldClim Dataset (www.worldclim.org/bioclim) (Supplementary Table S14). We downloaded 19 bioclimatic variables for the LIG (ca. 120,000–140,000 years BP)^50^, LGM (ca. 22,000 years BP), current conditions (average from years 1950 to 2000) and 2050 RCP8.5 (average from 2041 to 2060) from WorldClim (all 30 arc sec resolution, original 2.5 minute resolution of LGM were resampled to a spatial resolution of 30 arc sec by ArcGIS). Future climate data were accessed from Phase 5 of the Coupled Model Intercomparison Project (CIMP5)^51^, where we selected scenario 2050 RCP8.5, the highest emission scenario, so that we could forecast the warmest future environments for alpine species^2^. For LGM and 2050 which include multi-GCMs, the 19 bioclimatic variables were averaged for 4 GCMs for the LGM (Supplementary Table S7) and 17 GCMs for 2050 (Supplementary Table S8)^27,28,52^, standard deviations of multi-GCMs were also calculated (Supplementary Fig. S7)^26^.

To mitigate issues of multicollinearity in our models, we selected bioclimatic variables with lower correlations bioclimatic variables by using the variance inflation factor (VIF) statistics using Biodiversity R package in R version 3.1.2^25^. We retained bioclimatic variables with VIF values lower than 10 for all species records in each run, until all remaining VIF values were less than 10, as VIF values higher than 10 indicate strong collinearity^53,54^. Our final dataset included nine bioclimatic variables with VIF values lower than 10 (4 temperature-related variables (BIO2, BIO3, BIO8 and BIO9) and 5 precipitation variables (BIO13, BIO14 BIO15 BIO18 and BIO19)) (Supplementary Table S15).

### Modelling process

We used three methods in our modelling process: (1) MEAN ENSEMBLE (using each GCM to do ensemble ENM, and then calculating the mean and standard deviation of all predictions); (2) MMM ENSEMBLE (using the multi-model mean (MMM) of all GCMs to do ensemble ENM); (3) Maxent (using the MMM of all GCMs to do Maxent modelling). For each method, we did ENM for individual species, clade, distribution, and breeding system groups defined above (G_clade1_, G_clade2_, G_QTP_, G_HM_, G_wide_, G_plain_, G_dio_, G_mon_) and finally for all 14 species combined (WHOLE).

For the MEAN ENSEMBLE and MMM ENSEMBLE approaches, we undertook a three-step process in the BiodiversityR package^25^ to prepare for consensus mapping for each species and species group. The first step involved calibration of niche modelling algorithms, applying the ‘ensemble.test.splits’ function where we calibrated 17 ENM sub-models and defined a 4-fold cross-validation using 75% of the data to evaluate the remaining 25% data. AUC values for sub-models were used to determine their weights for ensemble models (Supplementary Table S1)^25^. For the second step we applied the “ensemble.test” function with sub-model weights >0.05 retained for ensemble modelling (Supplementary Table S16) – this used 10 internal test runs and 4-fold cross-validations predicting final weights for the sub-models. The last step was “ensemble.raster” function, which generated our consensus mapping. Upon completion of our MEAN ENSEMBLE models, we calculated the mean and standard deviation of all predictions.

AUC values of sub-models from the ensemble ENM indicated that Maxent had the highest performance among all the sub-models. Thus, we used Maxent to predict range shifts for *Rhodiola* species and species groups. We randomly selected 25% of the locations as test data and used the remaining 75% as the training dataset. This was the same approach used for ensemble modelling; we used default values for other parameters^30^.

### Estimating range shifts

We created a python script (see Supplementary Material2) to calculate the predicted suitable area, mean elevation, and mean center of the predicted distribution in ArcGIS for each model based on 25% habitat suitability. The predicted suitable area was calculated using rectangular projections. We calculated the mean elevation of each suitable area in meters, and found the mean center of the suitable area by calculating the mean longitude and latitude (for python script see Supplementary Material2).

The following equations were used to estimate the range shifts between different time periods:

1 $${\rm{\Delta }}S=(1-\frac{{{\rm{S}}}_{t{\rm{n}}+1}}{{{\rm{S}}}_{t{\rm{n}}}})/{\rm{\Delta }}{\rm{T}}$$

2 $$u=({V}_{t{\rm{n}}+1}-{V}_{t{\rm{n}}})/{\rm{\Delta }}{\rm{T}}$$

where for (1) ΔS is the range of distribution shift; S_t_ represents the suitable area at time ‘*t*’; *t*_n_ represents time for different periods (four periods in total: LIG, LGM, current, and future); ΔT equals the years from the periods *t*_n_ to *t*_n+1_, ΔT1 ≈ 98,000 (from LIG to LGM), ΔT2 ≈ 22,000 (LGM to current), ΔT3 ≈ 75 (current to future) and for (2) *u* equals the variation rate of elevation (*u*_alt_) or longitude and latitude of distribution center (*u*_lon_ and *u*_lat_) and *V*_*t*_ equals the mean elevation or mean center at time ‘*t*’.

### Data analysis

Wilcoxon rank sum tests were used to evaluate whether models differed in AUC values for models with all clades, breeding and distribution groups, individual species, and genus^49^.

Jackknife tests were used to measure variable importance^29^, with higher gain indicating a variable that contributes highly to the species’ spatial distribution. For a better understanding of the contributions of temperature and precipitation variables to the distribution of *Rhodiola* species, we first performed separate Principal Components Analysis (PCA) on the four temperature variables and five precipitation variables using records for all species (WHOLE)^49^. These ordinations produced two major temperature axes (Temp1 and Temp2) and two major precipitation axes (Prec1 and Prec2). To further reduce the dimensionality of the PCA data, we then used the scores of PC axes to do canonical discriminants analysis (CDA) (considering both temperature variables and precipitation variables) for G_QTP_, G_HM_, G_plain_ and G_wide_ (Fig. 2)^49^. Mean discriminant scores of species groups were plotted against the bioclimatic variables with the highest contributions to evaluate the relationship between known geographical distributions and climate variables (Supplementary Fig. S1). Wilks’ Lambda values were used to test the significance of our CDA results.

To test the influence of evolutionary history on range shifts induced by climate warming, we did χ^2^ tests on ΔS, *u*_alt_, *u*_lon_ and *u*_lat_ during the LGM to current and current to future between each sister species pair and between two clades (G_clade1_ and G_clade2_). Additionally, χ^2^ tests were used to compare G_mon_ and G_dio_, as well as between groups pairs from G_QTP_, G_HM_, G_plain_ and G_wide_ to examine the effects of breeding systems and ecological niche differentiation on the response to climate change. These analyses were performed using SPSS Statistics ver. 22.

## Electronic supplementary material

Supplementary Information
