# Supplementary material for: Response to climate change of montane herbaceous plants in the genus Rhodiola predicted by ecological niche modelling
Source: Sci Rep. 2018 Apr 12;8:5879. doi: 10.1038/s41598-018-24360-9 (PMC5897335; doi:10.1038/s41598-018-24360-9)
Supplement: Supplementary file 1 — Supplementary Information [file 41598_2018_24360_MOESM1_ESM.pdf]

Response to climate change of montane herbaceous plants in the genus *Rhodiola* predicted by ecological niche modelling

Jianling You[1], Xiaoping Qin[1], Sailesh Ranjitkar[2], Stephen C. Lougheed[3], Mingcheng Wang[2], Wen Zhou[1], Dongxin Ouyang[1], Yin Zhou[1], Jianchu Xu[2], Wenju Zhang[1], Yuguo Wang[1], Ji Yang[1] and Zhiping Song [1*]

Supplementary Table S1 Average AUC values of the sub-models used to determine the weights for the ensemble models of *Rhodiola* species and species groups.

	ENSEMBLE	MAXENT	RF	MGCV	GBMSTEP	GLMSTEP	MGCVFIX	GBM	FDA	MAHAL	SVM	EARTH	GAM	GAMSTEP	DOMAIN	SVME	NNET	BIOCLIM	RPART	GLM
R. alsia	0.983	0.981	0.982	0.980	0.975	0.976	0.977	0.975	0.978	0.971	0.972	0.975	0.970	0.967	0.974	0.950	0.960	0.876	0.782	0.507
R. bupleuroides	0.984	0.983	0.981	0.980	0.974	0.973	0.973	0.968	0.980	0.972	0.959	0.962	0.961	0.959	0.948	0.946	0.948	0.937	0.861	0.977
R. chryanthemifolia	0.991	0.990	0.991	0.990	0.985	0.990	0.986	0.985	0.985	0.988	0.968	0.986	0.974	0.973	0.973	0.935	0.931	0.908	0.901	0.529
R. crenulata	0.981	0.976	0.979	0.973	0.970	0.975	0.972	0.967	0.972	0.976	0.960	0.955	0.962	0.963	0.960	0.937	0.945	0.926	0.826	0.862
R. dumulosa	0.973	0.967	0.964	0.964	0.965	0.955	0.956	0.960	0.951	0.951	0.953	0.951	0.912	0.911	0.911	0.911	0.925	0.909	0.792	0.790
R. fastigiata	0.985	0.983	0.979	0.982	0.970	0.982	0.982	0.971	0.977	0.970	0.961	0.966	0.971	0.971	0.965	0.943	0.960	0.920	0.925	0.873
R. forrestii	0.994	0.993	0.991	0.993	0.989	0.991	0.987	0.987	0.992	0.991	0.957	0.905	0.981	0.982	0.990	0.906	0.930	0.850	0.846	0.604
R. henryi	0.995	0.995	0.991	0.995	0.986	0.994	0.990	0.987	0.984	0.991	0.991	0.956	0.964	0.963	0.984	0.952	0.956	0.928	0.945	0.900
R. himalensis	0.982	0.974	0.980	0.975	0.975	0.970	0.967	0.974	0.965	0.968	0.965	0.969	0.954	0.955	0.938	0.929	0.947	0.912	0.913	0.520
R. kirilowii	0.979	0.970	0.973	0.966	0.966	0.954	0.959	0.962	0.955	0.951	0.961	0.958	0.913	0.913	0.913	0.926	0.867	0.910	0.817	0.772
R. quadrifida	0.968	0.959	0.954	0.957	0.955	0.953	0.955	0.956	0.952	0.949	0.949	0.944	0.930	0.930	0.914	0.912	0.931	0.895	0.837	0.499
R. sacra	0.994	0.994	0.991	0.993	0.982	0.993	0.993	0.981	0.987	0.991	0.982	0.992	0.987	0.986	0.981	0.959	0.961	0.930	0.939	0.716
R. wallichiana	0.979	0.972	0.968	0.953	0.959	0.931	0.915	0.965	0.962	0.918	0.914	0.902	0.932	0.926	0.896	0.881	0.878	0.817	0.841	0.849
R. yunnanensis	0.991	0.990	0.988	0.989	0.985	0.990	0.989	0.982	0.988	0.990	0.987	0.862	0.972	0.972	0.983	0.977	0.959	0.947	0.900	0.989
G_{clade1}	0.986	0.984	0.975	0.980	0.972	0.979	0.976	0.973	0.980	0.971	0.944	0.972	0.954	0.955	0.959	0.953	0.945	0.942	0.917	0.761
G_{clade2}	0.970	0.964	0.972	0.966	0.969	0.966	0.967	0.961	0.957	0.955	0.961	0.960	0.915	0.915	0.897	0.950	0.943	0.919	0.916	0.905
G_{mon}	0.977	0.974	0.976	0.970	0.973	0.964	0.968	0.963	0.962	0.953	0.946	0.958	0.925	0.924	0.919	0.929	0.931	0.926	0.896	0.746
G_{dio}	0.973	0.968	0.973	0.970	0.972	0.970	0.971	0.967	0.964	0.957	0.967	0.963	0.922	0.922	0.906	0.954	0.933	0.925	0.934	0.970
G_{QTP}	0.977	0.974	0.978	0.973	0.977	0.972	0.974	0.975	0.969	0.965	0.970	0.969	0.950	0.950	0.941	0.959	0.949	0.944	0.948	0.973
G_{HM}	0.993	0.992	0.991	0.992	0.990	0.992	0.992	0.985	0.990	0.992	0.989	0.987	0.975	0.975	0.987	0.977	0.967	0.964	0.912	0.992
G_{wide}	0.975	0.971	0.971	0.969	0.966	0.965	0.966	0.957	0.956	0.955	0.959	0.956	0.927	0.926	0.927	0.935	0.921	0.922	0.874	0.744
G_{plain}	0.995	0.995	0.991	0.995	0.986	0.994	0.990	0.987	0.984	0.991	0.991	0.956	0.964	0.963	0.984	0.952	0.956	0.928	0.945	0.900
WHOLE	0.970	0.965	0.972	0.966	0.970	0.966	0.967	0.963	0.959	0.955	0.963	0.963	0.914	0.914	0.901	0.952	0.916	0.924	0.919	0.952
Average	0.982	0.979	0.979	0.977	0.974	0.974	0.973	0.972	0.972	0.968	0.964	0.955	0.949	0.948	0.946	0.940	0.937	0.916	0.886	0.797

Supplementary Table S2 Model evaluation metrics for Maxent: means test omission rates (OR) using the 10% training presence threshold, area under the receiver operating characteristic curve (AUC), true skill statistic (TSS) and test gains for all models of *Rhodiola* species.

	Threshold	OR	AUC	TSS	Test gain
R. alsia	0.378	0.116	0.979	0.956	2.675
R. bupleuroides	0.274	0.117	0.980	0.965	2.830
R. chryanthemifolia	0.378	0.109	0.988	0.982	3.333
R. crenulata	0.337	0.112	0.980	0.963	2.821
R. dumulosa	0.418	0.125	0.962	0.929	2.095
R. fastigiata	0.258	0.117	0.982	0.971	2.892
R. forrestii	0.456	0.148	0.993	0.985	3.639
R. henryi	0.292	0.121	0.991	0.989	3.550
R. himalensis	0.435	0.127	0.974	0.955	2.587
R. kirilowii	0.245	0.111	0.973	0.953	2.497
R. quadrifida	0.387	0.150	0.957	0.902	1.964
R. sacra	0.372	0.113	0.993	0.990	3.785
R. wallichiana	0.362	0.132	0.979	0.951	2.702
R. yunnanensis	0.298	0.117	0.989	0.985	3.322
G_{clade1}	0.291	0.114	0.982	0.978	2.958
G_{clade2}	0.288	0.103	0.929	0.923	1.621
G_{mon}	0.290	0.105	0.969	0.954	2.418
G_{dio}	0.292	0.102	0.932	0.927	1.677
G_{QTP}	0.276	0.099	0.948	0.941	1.962
G_{HM}	0.280	0.119	0.987	0.984	3.254
G_{wide}	0.287	0.114	0.966	0.949	2.314
G_{plain}	0.292	0.121	0.991	0.989	3.550
WHOLE	0.291	0.104	0.925	0.926	1.578

Supplementary Table S3 Percentage of variable contribution based on jackknife test. The highest contributions are presented in bold.

	Mean Diurnal Range (BIO2)	Isothermality (BIO3)	Mean Temperature of Wettest Quarter (BIO8)	Mean Temperature of Driest Quarter (BIO9)	Precipitation of Wettest Month (BIO13)	Precipitation of Driest Month (BIO14)	Precipitation Seasonality (BIO15)	Precipitation of Warmest Quarter (BIO18)	Precipitation of Coldest Quarter (BIO19)
R. alsia	0	26.7	**35.9**	18.2	0	0	0.8	11.7	6.7
R. bupleuroides	0.1	**36.6**	21.5	34.5	0.6	0.1	0.7	5.7	0.2
R. chryanthemifolia	0	43.8	3.1	**49.6**	0	0	0.8	0.7	2
R. crenulata	0.1	**41**	35.4	15	0.1	0	0	8.4	0.2
R. dumulosa	4.4	3.7	**36.3**	19.1	0.6	0.1	0.2	18.6	17.1
R. fastigiata	1.6	**37.4**	19.3	30.3	0.1	0	0.4	9.3	1.6
R. forrestii.	0	33.8	5.2	**44.9**	0.4	0.2	0	13.5	2.1
R. henryi.	17.8	0.2	6.2	**45.4**	0.3	0.8	4.8	20.2	4.4
R. himalensis	0.1	26.1	**37.3**	18.8	0	1.8	0	13.5	2.3
R. kirilowii	2.4	3.8	**31.6**	27.5	0.1	0.5	2.3	20.1	11.9
R. quadrifida	0	8.7	**66.5**	8.4	0.1	0.9	0.3	6.9	8.1
R. sacra	0.2	**40.3**	1	39	0	5.6	5.1	4.4	4.3
R. wallichiana	0.2	25.3	22.6	**50.6**	0	0.5	0	0.4	0.5
R. yunanensis	3.8	16.3	11	**37.3**	1.2	0.3	0.6	26.4	3.3
G_{clade1}	0.5	**38.9**	15.4	38.7	0.1	0	3	3.2	0.3
G_{clade2}	2.4	18.9	19.7	**36.6**	0.6	0.3	1	19.5	1.1
G_{mon}	3	14	27.1	**31.9**	2.4	1.8	2.4	15.9	1.6
G_{dio}	2.1	21	18.6	**37.2**	0.3	0.1	1	18.7	1
G_{QTP}	0.6	**40**	26.2	23.3	0.1	0.1	0.2	9.3	0.3
G_{HM}	2.6	16.7	13.1	**31.6**	1.1	0.3	0.5	30.8	3.4
G_{wide}	3	6.1	**30**	23.2	0.2	0.4	2.4	22.7	12
G_{plain}	17.8	0.2	6.2	**45.4**	0.3	0.8	4.8	20.2	4.4
WHOLE	2	21.4	19.1	**36.8**	0.9	0.2	1	17.9	0.9

Supplementary Table S4 Factor loadings of bioclimatic variables for temperature principal components analysis axes (Temp1 and Temp2).

	Temp1	Temp2
BIO2	-0.505	0.743
BIO3	0.090	0.979
BIO8	0.796	-0.378
BIO9	0.971	0.128

Supplementary Table S5 Factor loadings of bioclimatic variables for principal components analysis axes (Prec1 and Prec 2).

	Prec1	Prec2
BIO13	0.212	0.973
BIO14	0.865	0.357
BIO15	-0.854	-0.091
BIO18	0.182	0.978
BIO19	0.879	0.157

Supplementary Table S6 Factor loadings of bioclimatic variables for canonical structure of discriminant functions (Function1, Function2 and Function3).

	Function1	Function2	Function3
Temp2	0.758	0.606	-0.076
Prec1	-0.503	0.448	-0.173
Temp1	-0.439	0.827	-0.227
Prec2	0.127	0.587	0.795

Supplementary Table S7 Predicted suitable area (km^2) of *Rhodiola* species and species groups for each GCMs in LGM of ensemble models.

	CCSM4	MIROC-ESM	MPI-ESM-P	MIROC	min	mean	max
R. alsia	29544	28432	30038	28209	23545	29754	34474
R. bupleuroides	33452	34597	37638	35386	26580	34687	43697
R. chryanthemifolia	16634	16794	16135	19685	11379	16815	23083
R. crenulata	20372	26761	24529	27065	14863	24662	34292
R. dumulosa	11873	9461	11526	12007	5519	9319	17138
R. fastigiata	29258	27539	28151	28538	22160	28098	34764
R. forrestii	7167	7836	8418	8042	5004	7336	10741
R. henryi	10346	11540	11798	11749	8578	11019	13928
R. himalensis	30972	31250	29564	28619	22568	30835	37563
R. kirilowii	12289	9744	10937	10222	5464	9301	15965
R. quadrifida	25039	20546	21001	19933	12031	19659	31544
R. sacra	8222	9198	7393	7998	5814	7972	10602
R. wallichiana	5534	7691	6846	7069	3380	5744	10365
R. yunnanensis	21270	19045	19128	19066	14531	19182	24790
G_{clade1}	27505	29152	31660	31317	22304	28961	37852
G_{clade2}	106901	103180	101560	102393	86103	101676	120858
G_{mon}	45945	39545	43263	42009	32686	42180	53332
G_{dio}	99322	97966	94813	98149	83113	96342	112189
G_{QTP}	82479	81207	82265	80436	72517	81275	90553
G_{HM}	19106	17834	19286	18157	13956	18180	23265
G_{wide}	44223	41406	44621	41469	35885	42441	50313
G_{plain}	10346	11540	11798	11749	8578	11019	13928
WHOLE	126056	102697	103574	103947	90466	105768	126949

Supplementary Table S8 Predicted suitable area (km²) of *Rhodiola* species and species groups for each GCMs in 2050 RCP8.5 of ensemble models.

	ACCESS1-0	BCC-CSM1-1	CCSM4	CNRM-CM5	GFDL-CM3	GISS-E2-R	HadGEM2-AO	HadGEM2-CC	HadGEM2-ES	INMCM4	IPSL-CM5A-LR	MIROC-ESM-CHEM	MIROC-ESM	MIROC5	MPI-ESM-LR	MRI-CGCM3	NorESM1-M	min	mean	max
R. alsia	29418	29656	28492	28898	28841	28099	30104	27855	28586	29339	27391	27552	29230	28908	28298	30579	29199	23614	29261	34670
R. bupleuroides	35812	34104	34035	35275	33066	42347	36106	35847	36853	35116	35641	31807	32316	34991	31800	34682	33365	28228	34554	41754
R. chryanthemifolia	18073	17177	16894	17672	14893	15924	17633	18161	18139	15985	17492	16718	14798	17956	16184	17117	17789	12266	17072	21784
R. crenulata	20633	24563	25895	21209	29605	27467	26844	28129	26511	22559	28567	24150	25682	28010	26552	27109	25075	15640	25781	35513
R. dumulosa	8121	9606	9059	9360	9807	9576	8398	7683	8779	9119	9785	9787	10454	9700	8744	9840	9468	5544	8183	13131
R. fastigiata	29561	29551	27429	28392	30788	37722	28811	26889	29258	27406	25936	27777	27038	27649	30540	30597	27685	22286	28843	35524
R. forrestii	8837	8379	8186	8367	7811	7710	8387	8957	9031	8810	8311	8776	7895	8179	8859	8103	7575	5751	8113	11028
R. henryi	11314	10442	12505	9978	10725	13046	10230	13030	10621	10304	10839	10617	12545	10647	11052	13339	12007	8780	11119	13856
R. himalensis	28041	30404	30255	31597	29897	28698	28789	27101	28443	30552	28239	31002	27523	32415	30919	33519	29966	21432	30171	38117
R. kirilowii	8846	9726	8763	8798	9857	9512	9138	7731	8853	9514	9431	9634	10317	9966	8999	10147	9159	5776	8666	12781
R. quadrifida	20705	21296	21411	21663	21408	22069	21264	23849	22929	18611	17468	22821	20942	22470	18680	21733	23702	13774	20056	29502
R. sacra	8520	8677	8493	8296	8057	8169	8382	8339	8425	8805	7780	8631	8577	8485	8521	8196	8489	6104	8260	10713
R. wallichiana	7747	6619	7098	7447	8226	8263	6716	6453	6770	7569	6527	6497	7275	6829	6792	7655	7506	3522	6680	11289
R. yunnanensis	19127	19042	18157	18733	17378	19157	18979	18541	18229	18966	19058	20103	18008	18426	17716	19395	18361	15282	18587	22106
G_{clade1}	25499	27717	27732	30525	26160	27270	27603	28568	28682	27369	26740	28553	26905	29758	27394	29421	25600	22301	27446	33391
G_{clade2}	103406	101271	100943	100699	107194	104742	102611	102612	102710	124392	104632	104340	104200	106627	99406	103111	98565	88541	103019	120278
G_{mon}	37023	39792	37941	40536	39777	39442	37753	37314	39219	39858	36923	41157	38666	41481	38904	40833	38713	31002	38612	47409
G_{dio}	95860	95227	94356	95489	103553	98217	95850	96888	97686	98238	98586	98469	98965	99850	95889	96678	92625	84451	96534	111009
G_{QTP}	81020	76746	80366	81563	88233	82078	80180	83867	83307	85228	83908	82261	84412	81269	80046	79807	78515	71219	82687	91663
G_{HM}	20094	19165	18276	18961	17590	19160	19286	19049	24080	19277	18414	19609	18386	18174	18692	18365	18021	15510	18794	22710
G_{wide}	43857	42808	42639	43470	43911	43196	43951	40978	42904	42111	41691	43167	42400	45335	40992	46232	42554	37469	42687	48867
G_{plain}	11314	10442	12505	9978	10725	13046	10230	13030	10621	10304	10839	10617	12545	10647	11052	13339	12007	8780	11119	13856
WHOLE	101585	101635	94261	102019	113063	109542	99224	120260	120009	110426	97775	96728	99321	106205	100330	102558	100329	88455	104059	120090

Supplementary Table S9 The range of distribution shift of *Rhodiola* species and species groups at each period (Δ*S*, %/year) for MEAN ENSEMBLE, MMM ENSEMBLE and Maxent calculated by equation (1). Δ*S*1, Δ*S*2 and Δ*S*3 are the values at the period from LIG to LGM, LGM to current and current to future, respectively.

	MEAN ENSEMBLE			MMM ENSEMBLE			Maxent		
	Δ*S*1	Δ*S*2	Δ*S*3	Δ*S*1	Δ*S*2	Δ*S*3	Δ*S*1	Δ*S*2	Δ*S*3
R. alsia	0.000002	-0.000001	0.000033	0.000000	0.000005	0.000715	0.000029	0.000006	-0.000022
R. bupleuroides	0.000000	0.000000	0.000067	0.000000	0.000000	0.000191	0.000003	0.000017	0.007481
R. chryanthemifolia	-0.000001	0.000004	-0.000904	-0.000002	0.000010	-0.000173	0.000028	0.000021	0.012870
R. crenulata	-0.000001	-0.000007	0.003249	-0.000005	0.000017	0.004461	0.000176	-0.000007	0.001148
R. dumulosa	-0.000002	0.000001	-0.001961	-0.000004	0.000017	0.000675	-0.000005	0.000017	-0.000833
R. fastigiata	0.000000	-0.000002	0.000854	-0.000001	0.000000	0.003008	0.000056	0.000015	0.005114
R. forrestii	0.000002	0.000005	0.000051	0.000003	0.000001	0.000782	0.000014	-0.000008	0.011645
R. henryi	-0.000003	-0.000003	0.000941	-0.000004	-0.000002	0.003132	0.000010	-0.000011	-0.011592
R. himalensis	0.000001	0.000001	-0.000679	0.000000	0.000008	0.000553	-0.000001	0.000019	0.002542
R. kirilowii	-0.000002	0.000000	-0.000920	-0.000003	0.000008	-0.000096	0.000003	0.000009	0.001541
R. quadrifida	0.000000	0.000008	-0.001805	-0.000004	0.000044	-0.000528	0.000004	-0.000006	-0.003235
R. sacra	-0.000001	0.000001	0.000177	-0.000002	0.000004	0.001812	0.000031	0.000070	0.016760
R. wallichiana	0.000000	0.000011	-0.000909	0.000000	0.000012	0.000988	0.000006	0.000002	0.003441
R. yunnanensis	0.000001	-0.000002	0.000034	0.000000	0.000002	0.000170	0.000007	-0.000004	0.000403
G_{clade1}	-0.000001	-0.000002	-0.000202	-0.000001	-0.000001	0.000398	-0.000001	0.000036	0.013119
G_{clade2}	-0.000001	0.000000	0.000301	-0.000001	0.000000	0.001947	0.000002	0.000013	0.001150
G_{mon}	0.000000	-0.000006	0.000653	-0.000001	-0.000004	0.001454	-0.000006	0.000026	0.009068
G_{dio}	-0.000001	0.000000	0.000124	-0.000001	0.000000	0.002484	0.000004	0.000011	0.001243
G_{QTP}	0.000000	-0.000001	0.000484	0.000000	0.000000	0.000695	0.000002	0.000022	0.002754
G_{HM}	0.000002	0.000001	0.000156	0.000002	-0.000001	0.001652	0.000013	-0.000006	0.000671
G_{wide}	0.000000	0.000000	-0.000058	0.000000	0.000001	0.000444	-0.000002	0.000012	0.002672
G_{plain}	-0.000003	-0.000003	0.000941	-0.000004	-0.000002	0.003132	0.000010	-0.000011	-0.011592
WHOLE	-0.000001	0.000001	-0.000455	-0.000002	0.000004	-0.000632	0.000002	0.000014	0.001322

Supplementary Table S10 Elevation variation rate of *Rhodiola* species and species groups for MEAN ENSEMBLE, MMM ENSEMBLE and Maxent at each period (u_{alt}, m/year) calculated by using equation (2). $u_{alt}1$, $u_{alt}2$ and $u_{alt}3$ are the values of elevation variation rate from LIG to LGM, LGM to current and current to future, respectively.

	MEAN ENSEMBLE			MMM ENSEMBLE			Maxent		
	$u_{alt}1$	$u_{alt}2$	$u_{alt}3$	$u_{alt}1$	$u_{alt}2$	$u_{alt}3$	$u_{alt}1$	$u_{alt}2$	$u_{alt}3$
R. alsia	-0.001	0.001	0.030	0.000	-0.002	0.697	0.000	0.024	3.320
R. bupleuroides	0.000	0.000	0.573	0.000	0.001	0.276	0.003	0.043	4.560
R. chryanthemifolia	0.000	0.000	-0.139	0.000	0.002	-0.276	-0.002	0.042	7.640
R. crenulata	0.001	0.002	0.169	0.002	-0.002	-0.075	-0.001	0.020	2.280
R. dumulosa	-0.001	-0.002	-1.869	-0.002	0.001	-0.348	-0.002	0.040	12.160
R. fastigiata	0.000	0.001	0.319	0.000	0.000	0.948	-0.001	0.029	5.267
R. forrestii	0.000	0.000	0.305	0.000	0.000	1.599	-0.002	0.035	8.560
R. henryi	0.002	0.000	-0.334	0.002	0.002	-1.648	0.005	0.016	22.067
R. himalensis	-0.001	0.001	0.015	-0.001	0.000	0.279	-0.003	0.023	3.053
R. kirilowii	-0.001	-0.001	-0.568	-0.002	0.004	-1.036	-0.003	0.034	12.240
R. quadrifida	0.001	0.000	-1.088	0.001	0.001	-0.494	0.002	0.018	2.693
R. sacra	-0.001	0.000	0.141	-0.001	0.000	0.127	0.007	0.019	5.587
R. wallichiana	0.000	-0.001	-0.008	0.000	-0.002	-0.509	0.003	0.053	2.960
R. yunnanensis	0.000	0.000	0.634	0.000	-0.001	0.107	-0.015	0.050	6.573
G_{clade1}	0.000	-0.002	0.436	0.000	-0.001	0.877	0.004	0.041	5.413
G_{clade2}	0.001	0.000	0.019	0.001	0.000	0.447	-0.009	0.047	6.787
G_{mon}	-0.001	0.002	-0.586	-0.001	0.001	-0.776	-0.012	0.054	7.347
G_{dio}	0.001	0.000	0.030	0.001	0.000	-3.460	-0.007	0.049	6.573
G_{QTP}	0.000	-0.002	0.708	0.000	-0.001	0.295	-0.005	0.029	3.147
G_{HM}	0.001	0.000	0.615	0.000	0.000	0.903	-0.017	0.052	6.760
G_{wide}	0.001	0.000	-0.214	0.001	-0.001	0.275	-0.003	0.034	12.133
G_{plain}	0.002	0.000	-0.334	0.002	0.002	-1.648	0.005	0.016	22.067
WHOLE	0.001	-0.001	0.230	0.000	0.001	-1.268	-0.009	0.048	6.413

Supplementary Table S11 The variation rate of longitude (u_{lon}) of distribution center of *Rhodiola* species and species groups at each period (°/year) for MEAN ENSEMBLE, MMM ENSEMBLE and Maxent calculated by equation (2). $u_{lon}1$, $u_{lon}2$, and $u_{lon}3$ are the values at the period from LIG to LGM, LGM to current, and current to future, respectively.

	MEAN ENSEMBLE			MMM ENSEMBLE			Maxent		
	$u_{lon}1$	$u_{lon}2$	$u_{lon}3$	$u_{lon}1$	$u_{lon}2$	$u_{lon}3$	$u_{lon}1$	$u_{lon}2$	$u_{lon}3$
R. alsia	0.000002	0.000001	-0.001101	0.000002	-0.000002	-0.005315	-0.000033	-0.000015	-0.010150
R. bupleuroides	-0.000001	-0.000024	0.002845	-0.000001	-0.000023	0.000022	-0.000004	-0.000084	-0.007880
R. chryanthemifolia	-0.000004	0.000004	0.002968	-0.000006	0.000012	-0.012894	-0.000028	-0.000096	-0.023154
R. crenulata	-0.000011	-0.000004	0.000324	-0.000026	0.000063	0.001765	-0.000057	-0.000031	-0.001310
R. dumulosa	0.000000	0.000008	0.009844	0.000005	-0.000016	0.000814	0.000015	-0.000174	-0.052299
R. fastigiata	0.000004	-0.000027	-0.000318	-0.000005	0.000013	-0.016515	-0.000023	-0.000119	-0.025147
R. forrestii	-0.000007	-0.000026	0.003383	-0.000014	0.000005	-0.000836	0.000001	-0.000034	-0.020692
R. henryi	0.000002	-0.000008	0.002082	0.000001	-0.000001	-0.000043	-0.000030	-0.000014	-0.283755
R. himalensis	0.000012	-0.000019	0.001572	0.000012	-0.000018	-0.002581	0.000012	-0.000104	-0.016271
R. kirilowii	0.000010	-0.000012	0.003803	0.000017	-0.000040	0.001625	0.000070	-0.000232	-0.040661
R. quadrifida	0.000001	-0.000014	0.004069	0.000007	-0.000041	-0.001850	-0.000027	0.000008	-0.002401
R. sacra	0.000003	0.000002	-0.002482	0.000000	0.000015	0.004611	-0.000073	-0.000050	-0.011320
R. wallichiana	0.000013	0.000081	-0.000349	0.000001	0.000133	0.008876	-0.000052	0.000059	-0.003034
R. yunnanensis	-0.000004	-0.000001	-0.001917	-0.000007	0.000012	-0.007157	0.000016	-0.000063	-0.033610
G_{clade1}	-0.000001	-0.000033	0.003973	-0.000007	-0.000004	0.001427	0.000012	-0.000121	-0.017659
G_{clade2}	-0.000008	0.000007	-0.000029	-0.000009	0.000013	-0.006934	0.000039	-0.000212	-0.037623
G_{mon}	0.000010	-0.000037	0.002038	0.000001	0.000003	-0.001269	0.000064	-0.000242	-0.044743
G_{dio}	-0.000006	0.000010	0.000537	-0.000006	0.000010	0.005014	0.000029	-0.000220	-0.029046
G_{QTP}	-0.000003	0.000030	-0.005221	-0.000003	0.000033	-0.006344	0.000008	-0.000152	-0.008153
G_{HM}	-0.000001	0.000037	-0.012430	0.000003	0.000019	-0.022213	0.000014	-0.000034	-0.030984
G_{wide}	-0.000006	0.000004	0.002028	-0.000006	0.000004	-0.005852	0.000105	-0.000269	-0.052864
G_{plain}	0.000002	-0.000008	0.002082	0.000001	-0.000001	-0.000043	-0.000030	-0.000014	-0.283755
WHOLE	-0.000006	0.000019	-0.002269	-0.000003	0.000007	-0.002762	0.000041	-0.000219	-0.030994

Supplementary Table S12 The variation rate of latitude (u_{lat}) of distribution center of *Rhodiola* species and species groups at each period (°/year) for MEAN ENSEMBLE, MMM ENSEMBLE and Maxent calculated by equation (2). $u_{lat}1$, $u_{lat}2$, and $u_{lat}3$ are the values at the period from LIG to LGM, LGM to current, and current to future, respectively.

	MEAN ENSEMBLE			MMM ENSEMBLE			Maxent		
	$u_{lat}1$	$u_{lat}2$	$u_{lat}3$	$u_{lat}1$	$u_{lat}2$	$u_{lat}3$	$u_{lat}1$	$u_{lat}2$	$u_{lat}3$
R. alsia	0.00001	0.00000	-0.00175	0.00001	0.00000	-0.00032	0.00002	0.00000	0.00674
R. bupleuroides	0.00000	-0.00001	-0.00055	0.00000	-0.00001	-0.00043	-0.00003	0.00004	0.01399
R. chryanthemifolia	0.00000	0.00000	-0.00068	0.00000	0.00000	-0.00063	0.00000	0.00005	0.01535
R. crenulata	0.00000	-0.00001	0.00078	0.00000	0.00001	0.00318	0.00002	0.00002	0.00637
R. dumulosa	0.00001	0.00000	0.00568	0.00001	0.00000	-0.00019	0.00001	0.00005	0.00323
R. fastigiata	0.00000	-0.00001	0.00024	0.00000	0.00000	-0.00035	0.00001	0.00003	0.00988
R. forrestii	0.00000	0.00000	0.00085	0.00000	0.00000	0.00371	0.00000	0.00006	0.01903
R. henryi	0.00000	0.00000	-0.00021	0.00000	-0.00002	0.00584	0.00001	0.00009	0.05217
R. himalensis	0.00000	-0.00001	-0.00047	0.00000	0.00000	-0.00128	0.00001	0.00002	0.00802
R. kirilowii	0.00000	0.00001	0.00105	0.00001	0.00000	0.00131	0.00002	-0.00002	0.01081
R. quadrifida	-0.00001	0.00001	0.00767	-0.00001	0.00001	0.00559	0.00001	-0.00004	-0.00057
R. sacra	0.00000	0.00000	-0.00016	0.00000	0.00000	-0.00104	0.00000	-0.00001	0.00925
R. wallichiana	0.00000	-0.00001	-0.00025	0.00000	-0.00001	0.00180	-0.00001	-0.00005	0.01361
R. yunnanensis	0.00000	-0.00001	-0.00057	0.00000	0.00000	-0.00191	0.00001	0.00004	0.01226
G_{clade1}	0.00000	-0.00001	-0.00151	0.00000	0.00000	-0.00139	-0.00004	0.00005	0.01706
G_{clade2}	0.00000	0.00000	-0.00012	0.00000	0.00001	0.00138	0.00001	0.00003	0.01223
G_{mon}	0.00000	-0.00002	0.00023	0.00000	-0.00001	0.00172	0.00000	-0.00002	0.01519
G_{dio}	0.00000	0.00000	0.00004	0.00000	0.00000	0.00916	0.00001	0.00296	-0.84653
G_{QTP}	0.00000	0.00000	0.00001	0.00000	0.00000	-0.00096	0.00000	0.00004	0.01206
G_{HM}	0.00001	-0.00001	0.00019	0.00000	0.00000	0.00060	0.00001	0.00003	0.01511
G_{wide}	0.00000	-0.00001	0.00221	-0.00001	0.00002	-0.00653	0.00002	-0.00003	0.01076
G_{plain}	0.00000	0.00000	-0.00021	0.00000	-0.00002	0.00584	0.00001	0.00009	0.05217
WHOLE	0.00000	0.00001	-0.00383	0.00000	0.00001	-0.00289	0.00001	0.00003	0.01129

Supplementary Table S13 The *p*-values in ^{2} tests for ΔS, u_{alt} and u_{lat} between three pairs of sister species, G_{clade1} and G_{clade2}, G_{mon} and G_{dio}, and each pair of G_{QTP}, G_{HM}, G_{plain} and G_{wide} from LGM to current and to future. "-" stands for the comparison has opposite sign symbol.

Comparison	MEAN ENSEMBLE			MMM ENSEMBLE			Maxent		
	ΔS	u_{alt}	u_{lat}	ΔS	u_{alt}	u_{lat}	ΔS	u_{alt}	u_{lat}
R. alsia vs R. fastigiata	0.999	0.627	-	0.996	-	1.000	-	0.584	< 0.001
R. forrestii vs R. yunnanensis	-	0.667	-	0.999	-	-	0.184	0.736	-
R. henryi vs R. quadrifida	-	0.192	-	-	-	-	0.007	0.018	-
G_{clade1} *vs* G_{clade2}	-	-	-	-	-	-	0.565	0.801	0.878
G_{mon} *vs* G_{dio}	-	-	-	-	< 0.001	-	0.684	0.906	-
G_{QTP} *vs* G_{HM}	-	-	-	-	-	-	-	0.383	-
G_{QTP} *vs* G_{wide}	-	-	-	1.000	0.975	0.991	0.765	0.118	0.108
G_{QTP} *vs* G_{plain}	0.999	-	-	-	-	-	-	0.027	0.001
G_{HM} *vs* G_{wide}	-	-	0.997	-	-	-	-	0.488	-
G_{HM} *vs* G_{plain}	-	-	-	0.998	-	-	-	0.18	-
G_{wide} *vs* G_{plain}	-	0.741	-	-	-	-	-	0.519	0.21

Supplementary Table S14 The 19 bioclimatic variables. Data collected from the WorldClim Dataset (www.worldclim.org/bioclim).

Code	Name of variable
BIO1	Annual Mean Temperature
BIO2	Mean Diurnal Range (Mean of monthly (max temp - min temp))
BIO3	Isothermality (BIO2/BIO7) (* 100)
BIO4	Temperature Seasonality (standard deviation *100)
BIO5	Max Temperature of Warmest Month
BIO6	Min Temperature of Coldest Month
BIO7	Temperature Annual Range (BIO5-BIO6)
BIO8	Mean Temperature of Wettest Quarter
BIO9	Mean Temperature of Driest Quarter
BIO10	Mean Temperature of Warmest Quarter
BIO11	Mean Temperature of Coldest Quarter
BIO12	Annual Precipitation
BIO13	Precipitation of Wettest Month
BIO14	Precipitation of Driest Month
BIO15	Precipitation Seasonality (Coefficient of Variation)
BIO16	Precipitation of Wettest Quarter
BIO17	Precipitation of Driest Quarter
BIO18	Precipitation of Warmest Quarter
BIO19	Precipitation of Coldest Quarter

Supplementary Table S15 The 9 bioclimatic variables with all the VIF values lower than 10 after ten test runs.

BIO5	BIO6	BIO7	BIO11	BIO4	BIO10	BIO1	BIO16	BIO13	BIO12	BIO17	BIO14	BIO3	BIO2	BIO9	BIO18	BIO8	BIO19	BIO15
Inf	Inf	Inf	8987.83	2700.59	1948.05	1431.19	273.84	140.18	112.98	62.29	49.58	29.76	28.09	18.14	17.63	8.83	7.37	4.47
	BIO11	BIO4	BIO10	BIO1	BIO6	BIO7	BIO16	BIO13	BIO12	BIO17	BIO14	BIO3	BIO2	BIO9	BIO18	BIO8	BIO19	BIO15
	8987.83	2700.59	1948.05	1431.19	1049.32	443.98	273.84	140.18	112.98	62.29	49.58	29.76	28.09	18.14	17.63	8.83	7.37	4.47
		BIO1	BIO10	BIO6	BIO4	BIO7	BIO16	BIO13	BIO12	BIO17	BIO14	BIO3	BIO2	BIO9	BIO18	BIO8	BIO19	BIO15
		1033.72	1026.10	920.03	522.74	443.97	272.80	140.17	112.22	62.24	49.31	28.04	27.75	18.07	17.57	8.68	7.26	4.30
			BIO6	BIO7	BIO10	BIO16	BIO4	BIO13	BIO12	BIO17	BIO14	BIO2	BIO3	BIO9	BIO18	BIO8	BIO19	BIO15
			889.67	443.44	282.43	271.44	240.53	137.61	112.19	61.22	47.43	26.18	25.71	18.05	17.11	8.17	7.20	4.20
				BIO16	BIO7	BIO4	BIO13	BIO12	BIO17	BIO14	BIO2	BIO3	BIO10	BIO9	BIO18	BIO8	BIO19	BIO15
				266.10	233.23	206.77	137.26	111.64	61.17	47.39	25.86	25.54	18.20	17.31	14.75	8.16	7.18	3.70
					BIO7	BIO4	BIO17	BIO12	BIO14	BIO13	BIO2	BIO3	BIO10	BIO9	BIO18	BIO8	BIO19	BIO15
					232.20	206.12	60.14	56.35	47.39	29.54	25.86	25.53	18.19	17.29	14.39	8.14	6.99	3.69
						BIO17	BIO12	BIO14	BIO13	BIO4	BIO3	BIO10	BIO9	BIO2	BIO18	BIO8	BIO19	BIO15
						59.96	52.31	47.39	28.92	27.12	23.98	18.14	17.28	9.90	9.11	8.10	6.87	3.69
							BIO12	BIO13	BIO4	BIO3	BIO10	BIO9	BIO2	BIO18	BIO8	BIO14	BIO19	BIO15
							52.07	28.85	27.10	23.89	17.88	17.27	9.85	9.06	7.78	6.78	5.97	3.60
								BIO4	BIO3	BIO10	BIO9	BIO2	BIO8	BIO13	BIO14	BIO18	BIO19	BIO15
								26.69	23.89	17.85	17.20	9.85	7.78	6.49	6.31	6.02	5.59	2.99
									BIO10	BIO9	BIO8	BIO13	BIO3	BIO14	BIO19	BIO18	BIO2	BIO15
									15.75	13.93	7.38	6.48	6.06	5.95	5.59	5.42	4.00	2.75
										BIO13	BIO14	BIO19	BIO18	BIO9	BIO2	BIO3	BIO15	BIO8
										6.43	5.92	5.57	5.42	3.44	3.22	3.20	2.73	1.92

Supplementary Table S16 Average weights for the ensemble models of *Rhodiola* species and species groups.

	MAXENT	MGCV	RF	GLMSTEP	GBMSTEP	MGCVFIX	GBM	GLM	MAHAL	FDA	EARTH	SVM	DOMAIN	GAM	GAMSTEP	NNET	RPART	SVME	BIOCLIM
R. alsia	0.143	0.107	0.143	0.071	0.071	0.072	0.071	0.000	0.071	0.107	0.071	0.071	0.000	0.000	0.000	0.000	0.000	0.000	0.000
R. bupleuroides	0.124	0.124	0.123	0.057	0.123	0.090	0.090	0.123	0.000	0.091	0.000	0.056	0.000	0.000	0.000	0.000	0.000	0.000	0.000
R. chryanthemifolia	0.130	0.130	0.129	0.130	0.065	0.096	0.065	0.000	0.097	0.065	0.095	0.000	0.000	0.000	0.000	0.000	0.000	0.000	0.000
R. crenulata	0.141	0.112	0.143	0.141	0.000	0.112	0.101	0.111	0.141	0.000	0.000	0.000	0.000	0.000	0.000	0.000	0.000	0.000	0.000
R. dumulosa	0.126	0.125	0.125	0.089	0.126	0.099	0.125	0.000	0.098	0.000	0.088	0.000	0.000	0.000	0.000	0.000	0.000	0.000	0.000
R. fastigiata	0.126	0.126	0.124	0.126	0.067	0.126	0.102	0.090	0.056	0.058	0.000	0.000	0.000	0.000	0.000	0.000	0.000	0.000	0.000
R. forrestii	0.129	0.129	0.097	0.097	0.097	0.065	0.097	0.000	0.129	0.097	0.000	0.000	0.065	0.000	0.000	0.000	0.000	0.000	0.000
R. henryi	0.130	0.130	0.097	0.129	0.097	0.065	0.097	0.065	0.065	0.062	0.000	0.064	0.000	0.000	0.000	0.000	0.000	0.000	0.000
R. himalensis	0.104	0.104	0.107	0.075	0.103	0.078	0.102	0.000	0.098	0.076	0.077	0.077	0.000	0.000	0.000	0.000	0.000	0.000	0.000
R. kirilowii	0.135	0.101	0.134	0.096	0.102	0.069	0.101	0.000	0.067	0.000	0.097	0.099	0.000	0.000	0.000	0.000	0.000	0.000	0.000
R. quadrifida	0.125	0.090	0.091	0.122	0.092	0.121	0.093	0.000	0.087	0.057	0.055	0.067	0.000	0.000	0.000	0.000	0.000	0.000	0.000
R. sacra	0.104	0.138	0.104	0.138	0.069	0.104	0.103	0.000	0.103	0.000	0.138	0.000	0.000	0.000	0.000	0.000	0.000	0.000	0.000
R. wallichiana	0.134	0.098	0.099	0.098	0.072	0.063	0.072	0.063	0.000	0.071	0.000	0.000	0.000	0.101	0.066	0.063	0.000	0.000	0.000
R. yunnanensis	0.117	0.116	0.115	0.116	0.056	0.083	0.056	0.083	0.117	0.083	0.000	0.000	0.060	0.000	0.000	0.000	0.000	0.000	0.000
G_{clade1}	0.129	0.128	0.085	0.127	0.120	0.000	0.101	0.062	0.000	0.127	0.121	0.000	0.000	0.000	0.000	0.000	0.000	0.000	0.000
G_{clade2}	0.097	0.129	0.130	0.129	0.129	0.129	0.096	0.097	0.000	0.000	0.064	0.000	0.000	0.000	0.000	0.000	0.000	0.000	0.000
G_{mon}	0.121	0.119	0.121	0.087	0.122	0.117	0.087	0.064	0.000	0.053	0.055	0.055	0.000	0.000	0.000	0.000	0.000	0.000	0.000
G_{dio}	0.119	0.120	0.120	0.120	0.120	0.120	0.054	0.120	0.000	0.053	0.000	0.054	0.000	0.000	0.000	0.000	0.000	0.000	0.000
G_{QTP}	0.129	0.129	0.129	0.097	0.129	0.129	0.129	0.129	0.000	0.000	0.000	0.000	0.000	0.000	0.000	0.000	0.000	0.000	0.000
G_{HM}	0.129	0.129	0.099	0.129	0.099	0.129	0.000	0.129	0.096	0.063	0.000	0.000	0.000	0.000	0.000	0.000	0.000	0.000	0.000
G_{wide}	0.136	0.134	0.136	0.133	0.132	0.133	0.106	0.000	0.000	0.000	0.090	0.000	0.000	0.000	0.000	0.000	0.000	0.000	0.000
G_{plain}	0.130	0.130	0.097	0.129	0.097	0.065	0.097	0.065	0.065	0.062	0.000	0.064	0.000	0.000	0.000	0.000	0.000	0.000	0.000
WHOLE	0.129	0.129	0.130	0.129	0.129	0.129	0.064	0.097	0.000	0.000	0.000	0.064	0.000	0.000	0.000	0.000	0.000	0.000	0.000
Average	0.125	0.121	0.116	0.111	0.096	0.095	0.087	0.056	0.056	0.049	0.041	0.029	0.005	0.004	0.003	0.003	0.000	0.000	0.000

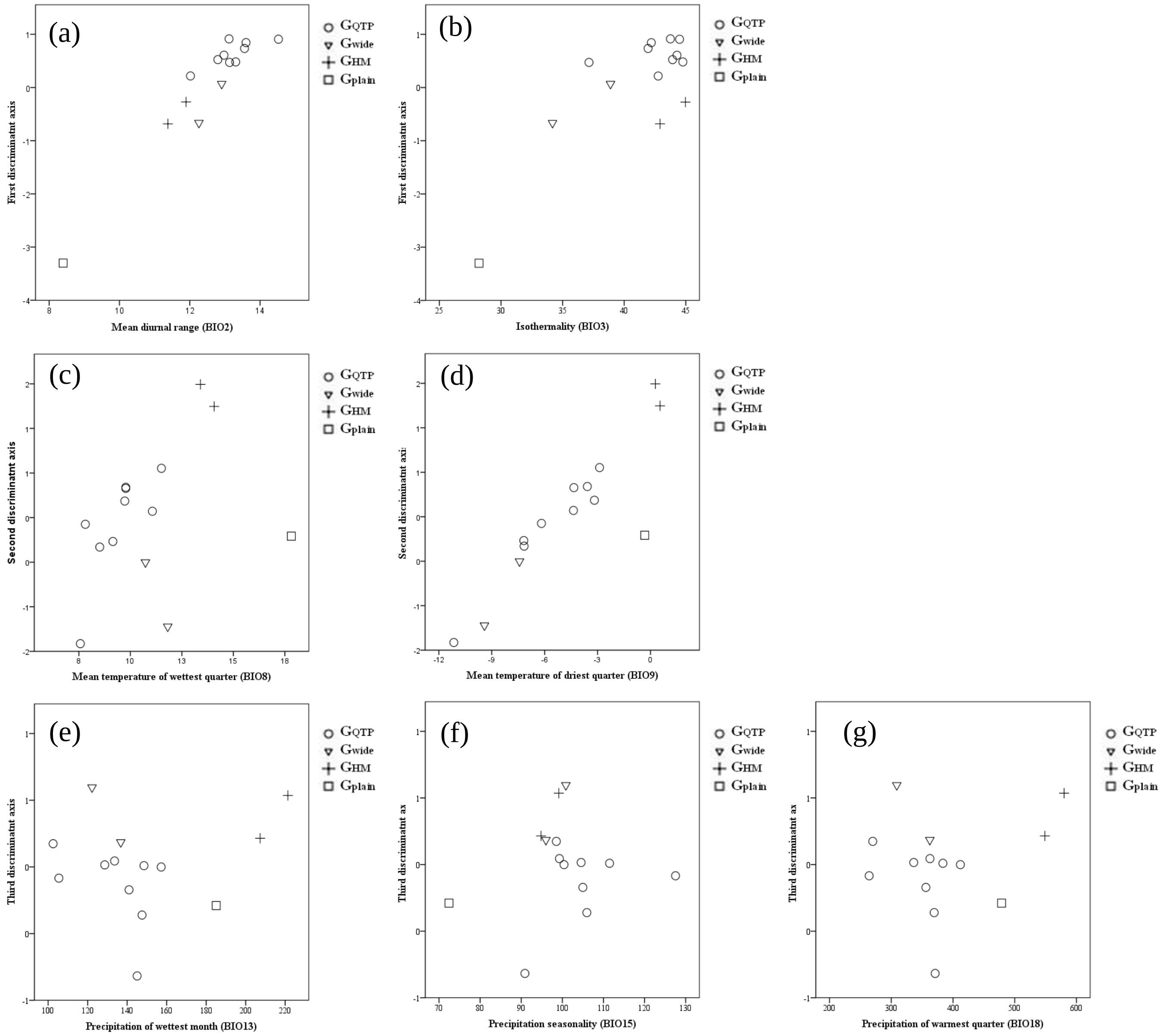

Supplementary Fig. S1. Mean species CDA scores versus bioclimatic variable plots. The relationship between (a) the first discriminant axis and mean diurnal range (BIO2), (b) the first discriminant axis and isothermality (BIO3), (c) the second discriminant axis and mean temperature of wettest quarter (BIO8), (d) the second discriminant axis and mean temperature of driest quarter (BIO9), (e) the third discriminant axis and precipitation of wettest month (BIO13), (f) the third discriminant axis and precipitation seasonality (BIO15), (g) the third discriminant axis and precipitation of warmest quarter (BIO18) for G_{QTP} (○), G_{wide} (▽), G_{HM} (┼) and G_{plain} (□).

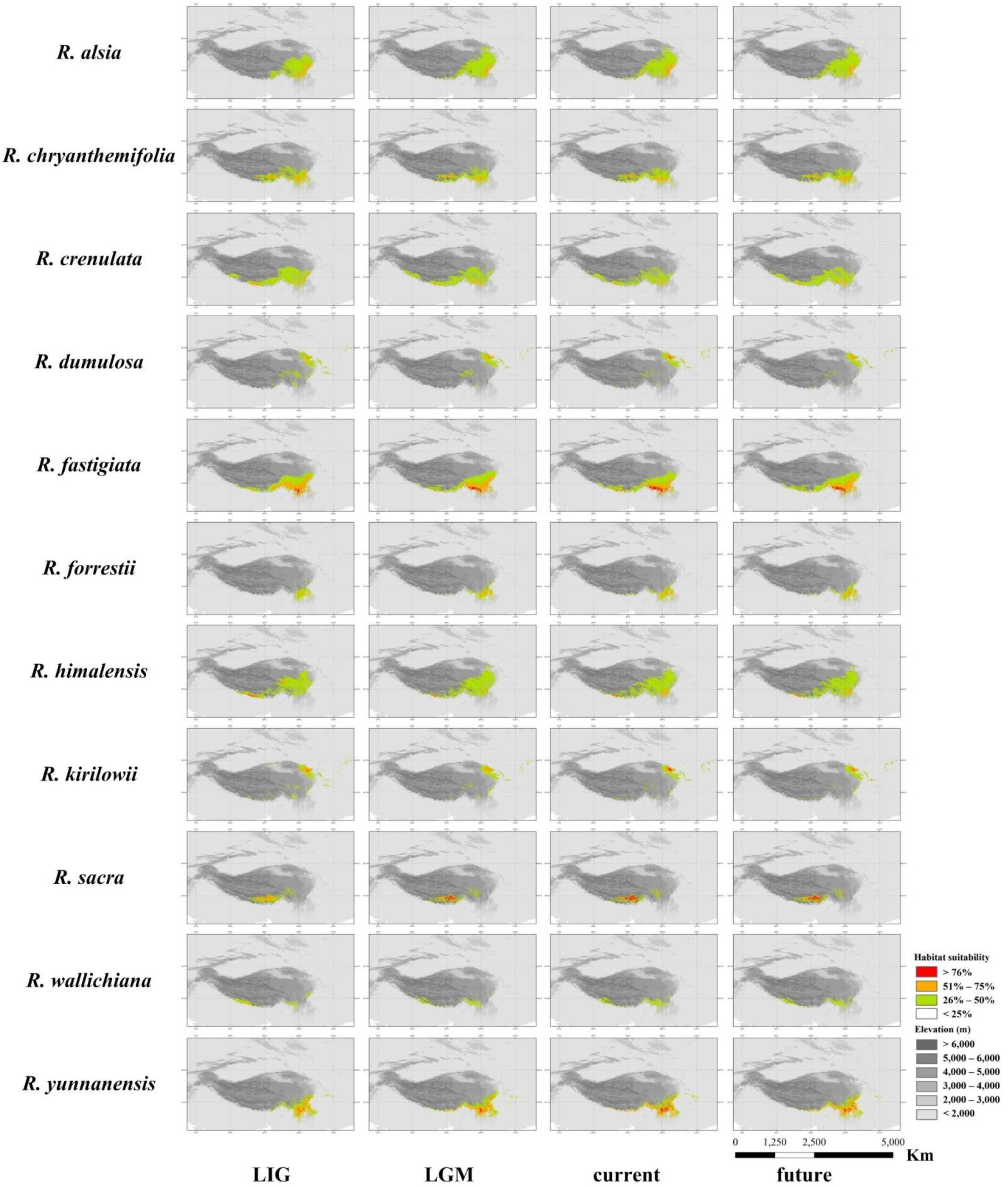

Supplementary Fig. S2 MEAN ENSEMBLE predicted maps for *Rhodiola* species and species group (except for those showed in Fig. 3) for the period LIG, LGM, current and future (2050). ENM predicted results were processed by ArcGIS ver 10.2 (ESRI, Redlands, California, USA) (http://www.esri.com/), and then integrated using Microsoft Office Visio 2013 (http://office.microsoft.com/visio/).

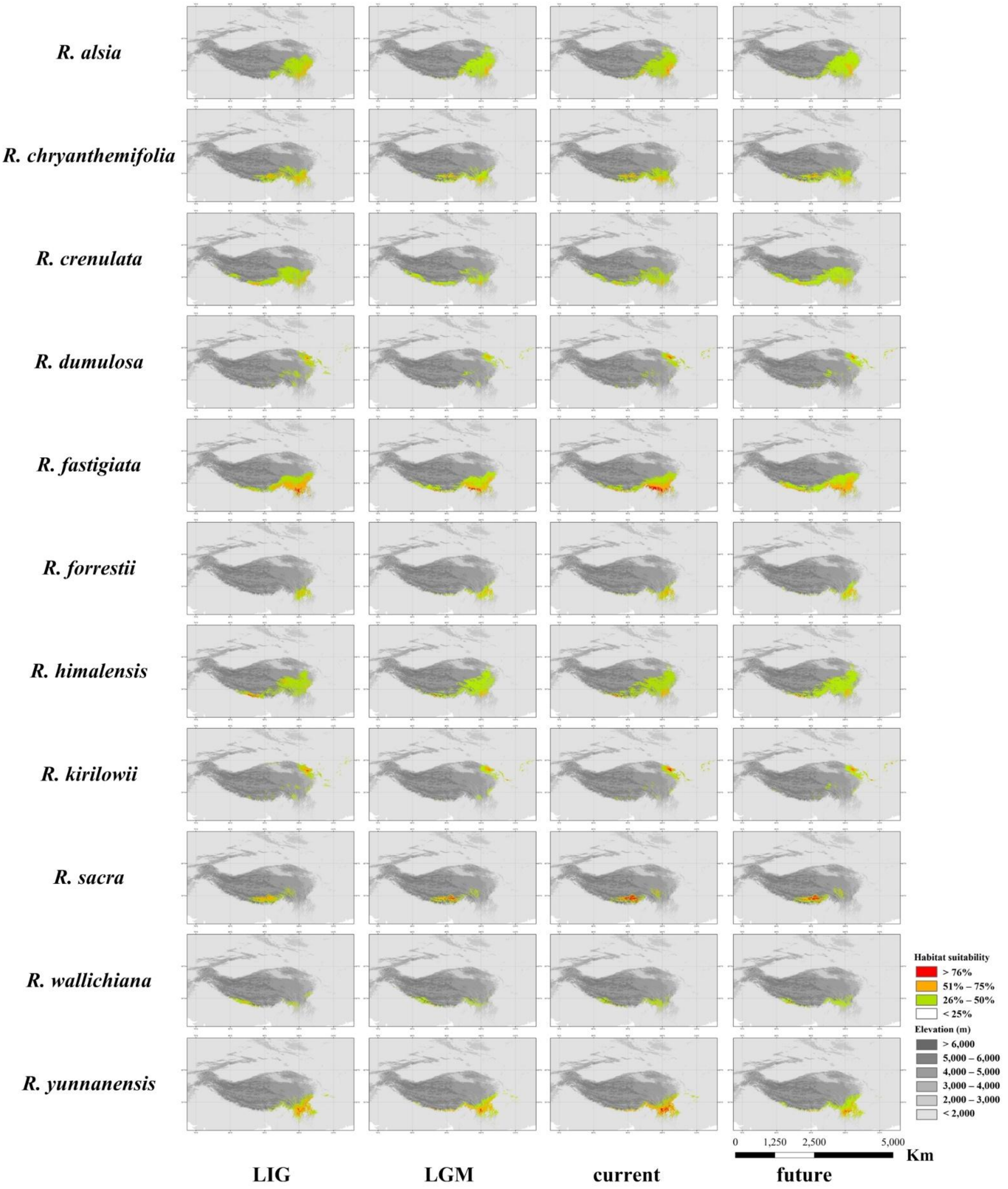

R. alsia
R. chryanthemifolia
R. crenulata
R. dumulosa
R. fastigiata
R. forrestii
R. himalensis
R. kirilowii
R. sacra
R. wallichiana
R. yunnanensis
LIG
LGM
current
future
Habitat suitability
> 76%
51% – 75%
26% – 50%
< 25%
Elevation (m)
> 6,000
5,000 – 6,000
4,000 – 5,000
3,000 – 4,000
2,000 – 3,000
< 2,000
0
1,250
2,500
5,000
Km

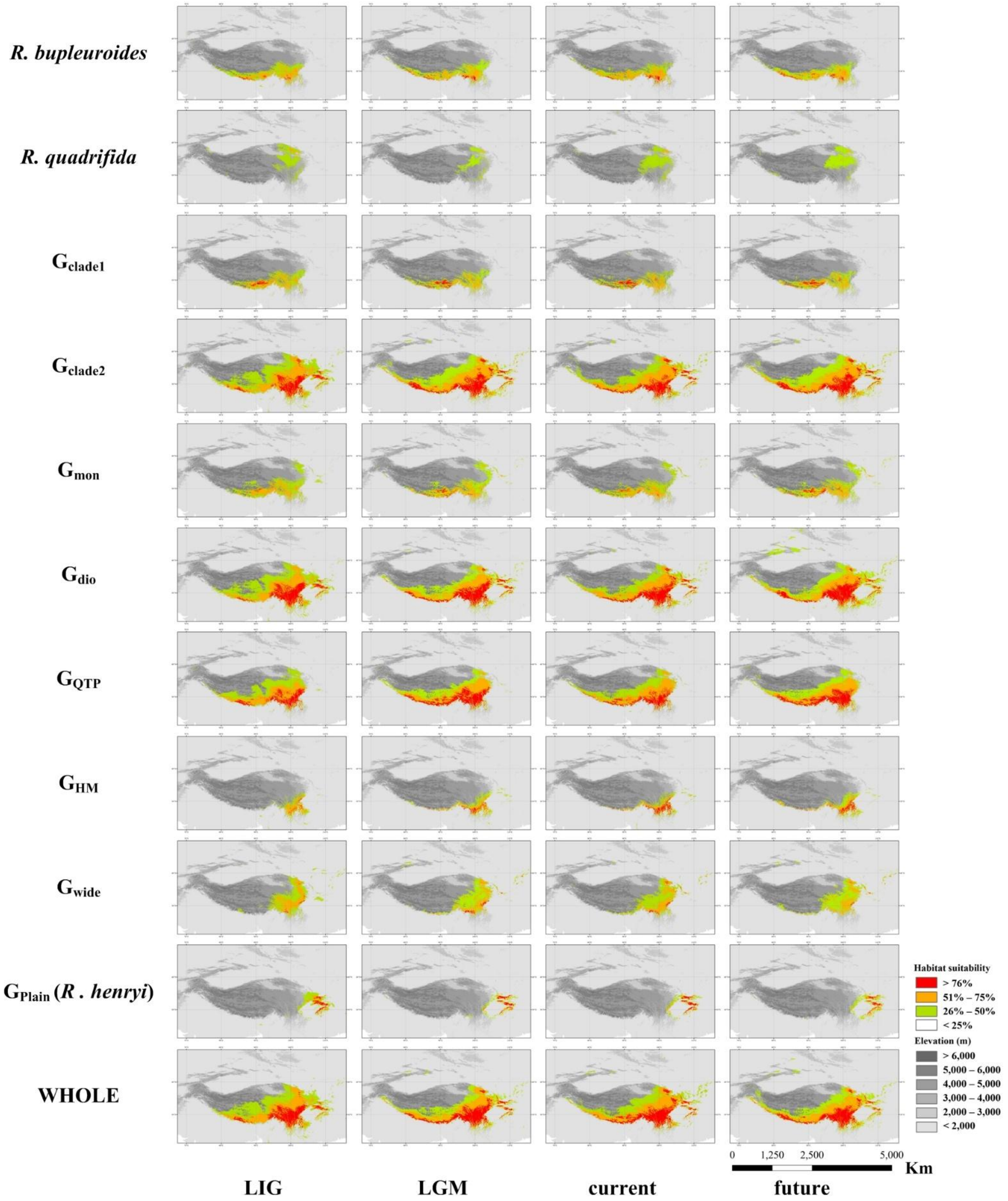

Supplementary Fig. S3 MMM ENSEMBLE predicted maps for each *Rhodiola* species and species group for the period LIG, LGM, current and future (2050). ENM predicted results were processed by ArcGIS ver 10.2 (ESRI, Redlands, California, USA) (http://www.esri.com/), and then integrated using Microsoft Office Visio 2013 (http://office.microsoft.com/visio/).

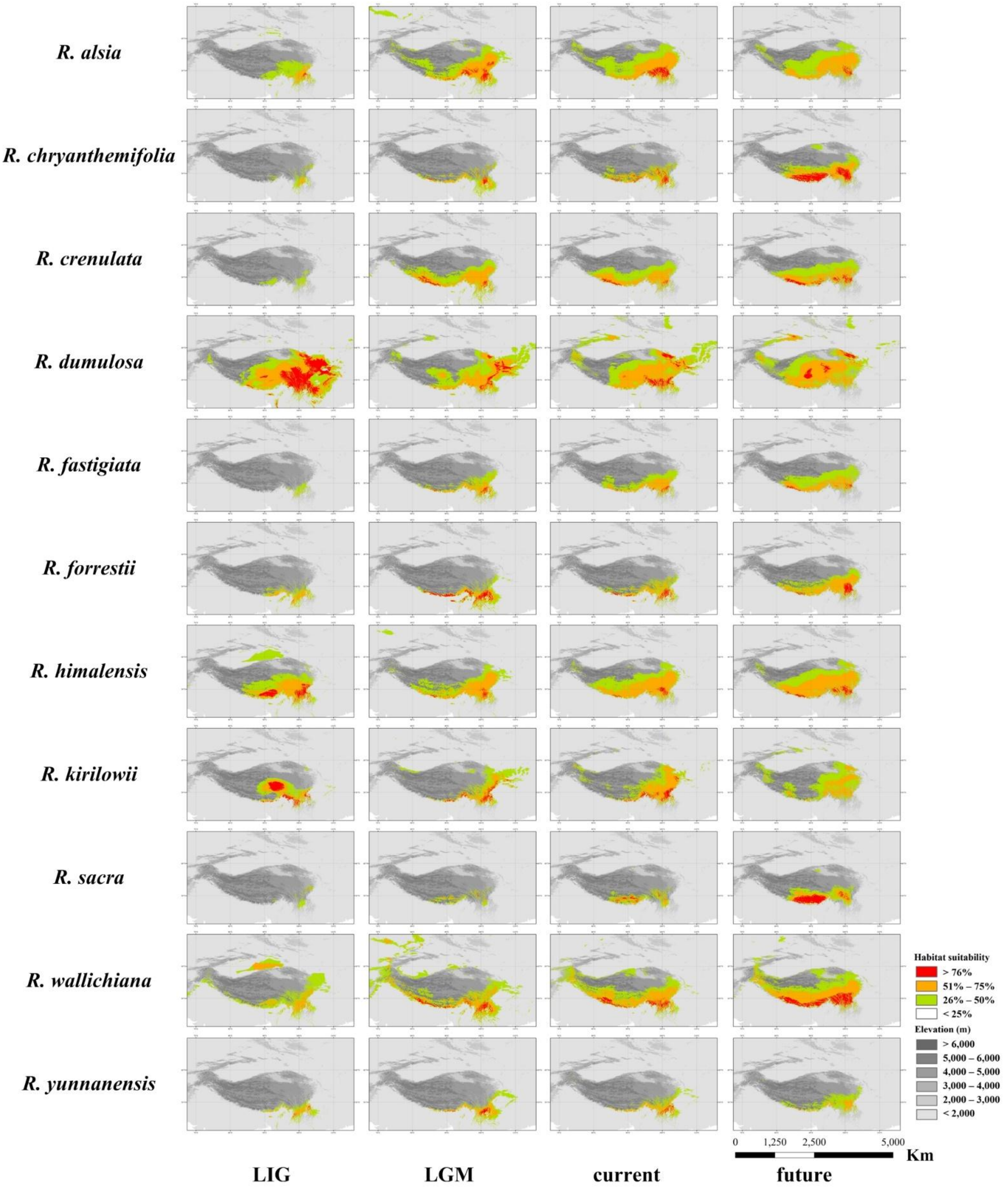
R. alsia
R. chryanthemifolia
R. crenulata
R. dumulosa
R. fastigiata
R. forrestii
R. himalensis
R. kirilowii
R. sacra
R. wallichiana
R. yunnanensis
Habitat suitability
> 76%
51% – 75%
26% – 50%
< 25%
Elevation (m)
> 6,000
5,000 – 6,000
4,000 – 5,000
3,000 – 4,000
2,000 – 3,000
< 2,000
0
1,250
2,500
5,000
Km
LIG
LGM
current
future

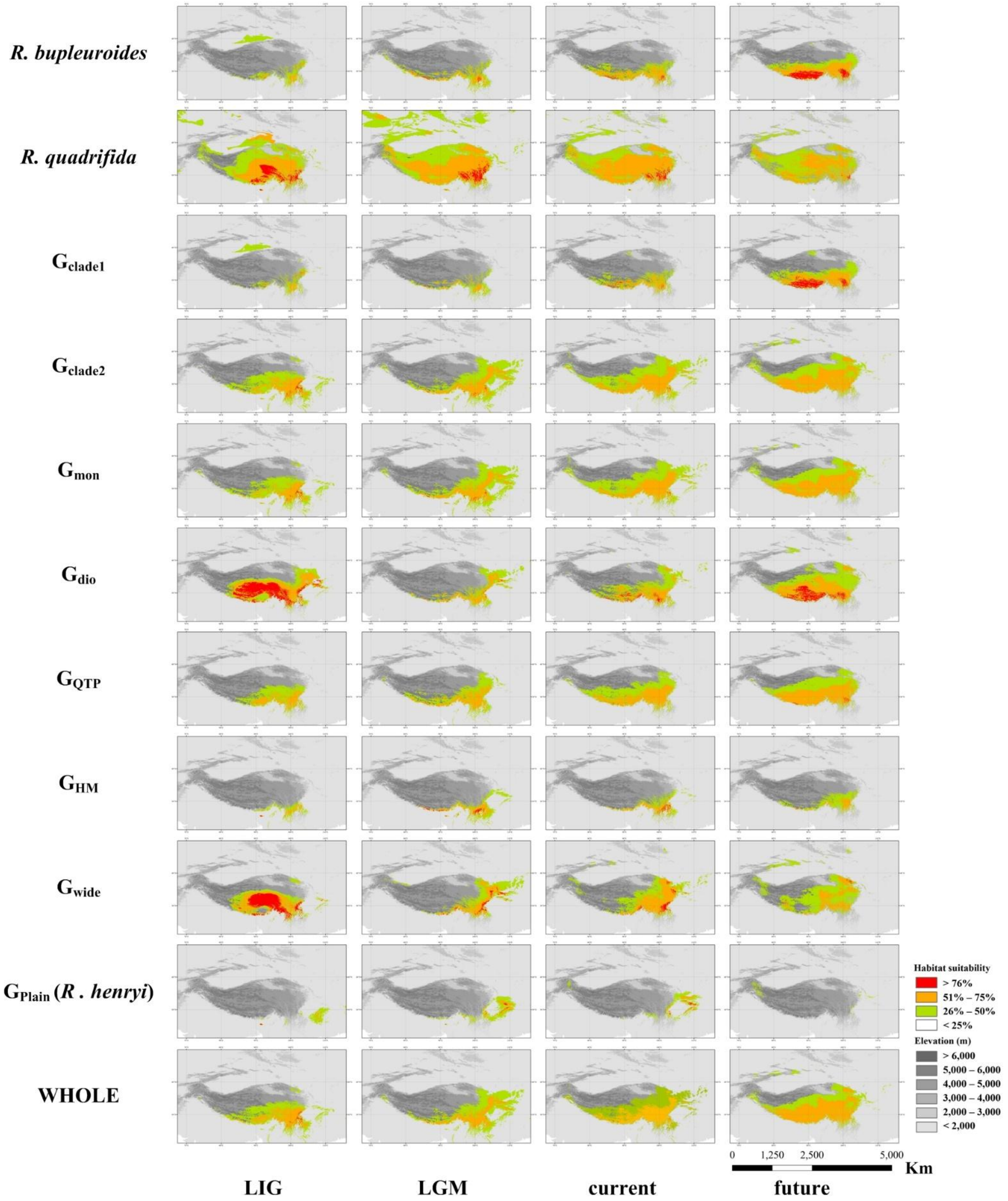

Supplementary Fig. S4 **Maxent predicted maps for each *Rhodiola* species and species group for the period LIG, LGM, current and future (2050). ENM predicted results were processed by ArcGIS ver 10.2 (ESRI, Redlands, California, USA) (http://www.esri.com/), and then integrated using Microsoft Office Visio 2013 (http://office.microsoft.com/visio/).**

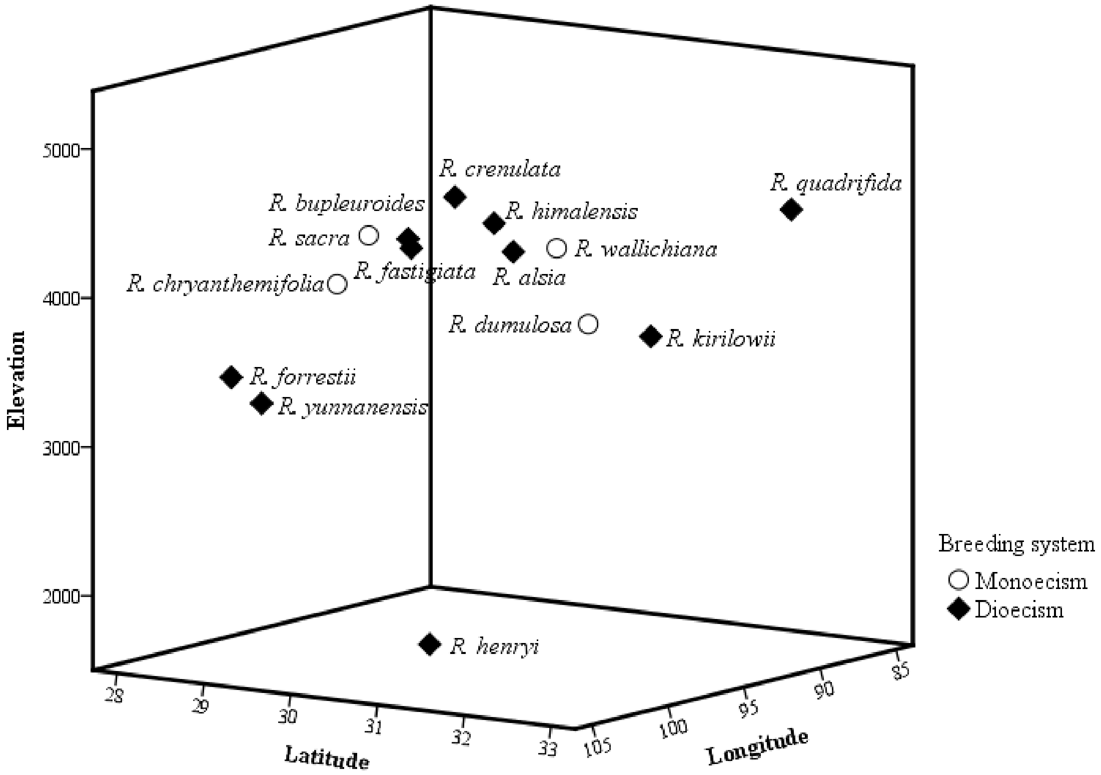

Supplementary Fig. S5. Mean elevation, latitude and longitude of 14 *Rhodiola* species that well modelled. "○" represents monoecism and "◆" represents dioecism.

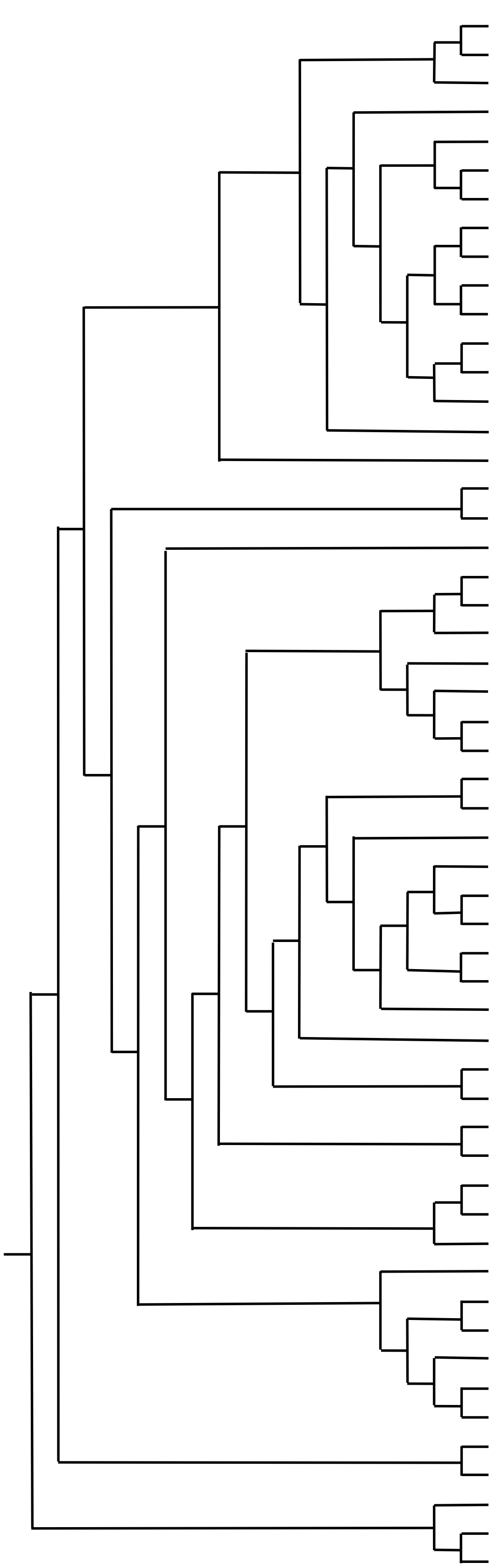

		CVH	GBIF	Ref	Field records	Sum
○	*R. semenovii*	0	0			0
○	*R. rhodantha*	0	173			173
◆	*R. integrifolia*	1	1046			1047
○	*R. wallichiana**	41	47			88
○	*R. hobsonii*	26	13			39
○	*R. ovatisepala*	20	27			47
○	*R. prainii*	8	23			31
○	*R. alterna*	17	7			24
○	*R. sacra v sacra**	69	58			127
○	*R. sexifolia*	28	4			32
◆	*R. stapfii*	13	7			20
○	*R. sinuata*	19	24			43
○	*R. chrysanthemifolia**	81	52	10	8	151
○	*R. humilis*	14	19			33
○	*R. liciae*	1	1			2
○	*R. smithii*	0	35			35
	Pseudosedum sp					
	Pseudosedum lievenii					
◆	*R. purpureoviridis*	98	38			136
◆	*R. yunnanensis**	530	168		7	705
◆	*R. forrestii**	109	22			131
◆	*R. atsaensis*	0	5			5
◆	*R. serrata*	7	5			12
◆	*R. bupleuroides**	263	167		32	462
◆	*R. calliantha*	4	0			4
◆	*R. discolor*	152	0			152
◆	*R. tibetica*	68	222			290
◆	*R. sachalinensis*	68	1			69
◆	*R. coccineae*	3	0			3
◆	*R. gannanica*	0	0			0
◆	*R. alsia**	95	35	18		148
◆	*R. fastigiata**	426	91	21	20	558
◆	*R. tangutica*	198	6			204
◆	*R. kirilowii**	558	117	29	3	707
○	*R. dumulosa**	362	44	43	2	451
◆	*R. scabrida*	73	24			97
◆	*R. crenulata**	164	58	10		232
○	*R. atuntsuensis*	12	10			22
◆	*R. quadrifida**	385	24			409
◆	*R. henryi**	557	3			560
◆	*R. rosea*	208	12835	113		13156
◆	*R. litwinowii*	3	0			3
◆	*R. heterodonta*	33	206			239
◆	*R. gelida*	97	37			134
◆	*R. nobilis*	33	58			91
◆	*R. humalensis**	201	60			261
◆	*R. brevipetiolata*	12	1			13
◆	*R. macrocarpa*	56	47			103
◆	*R. eurycarpa*	229	16			245
	Phedimus aizoon					
	Phedimus kamtschaticus					
	Hylotelephium tatarinowii					
	Sedum obtrullatum					
	Sedum oreades					

Supplementary Fig. S6. Phylogenetic tree of the genus *Rhodiola* modified from Zhang *et al*.[1] and the records of *Rhodiola* species. Including records from online herberium, references and our field records. (*, the species used for ENM; ○, monoecism; ◆, dioecism)

Standard deviations of multi-GCMs in LGM:

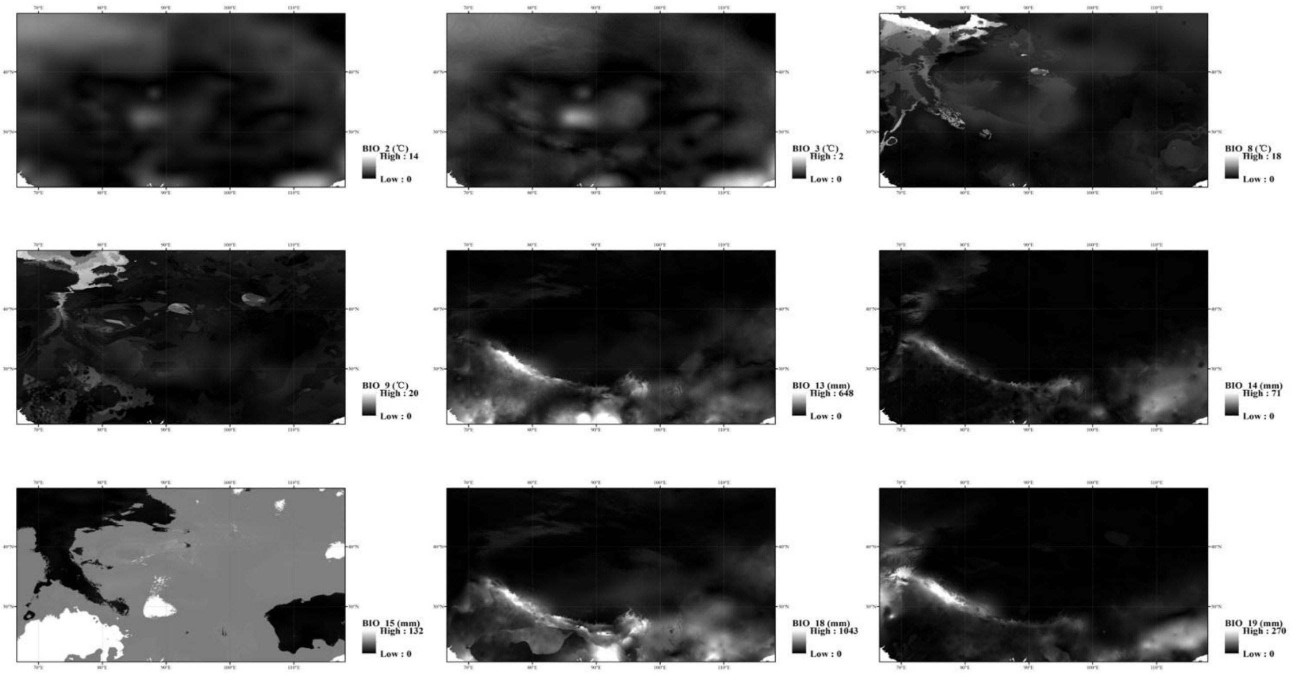

Standard deviations of multi-GCMs in 2050:

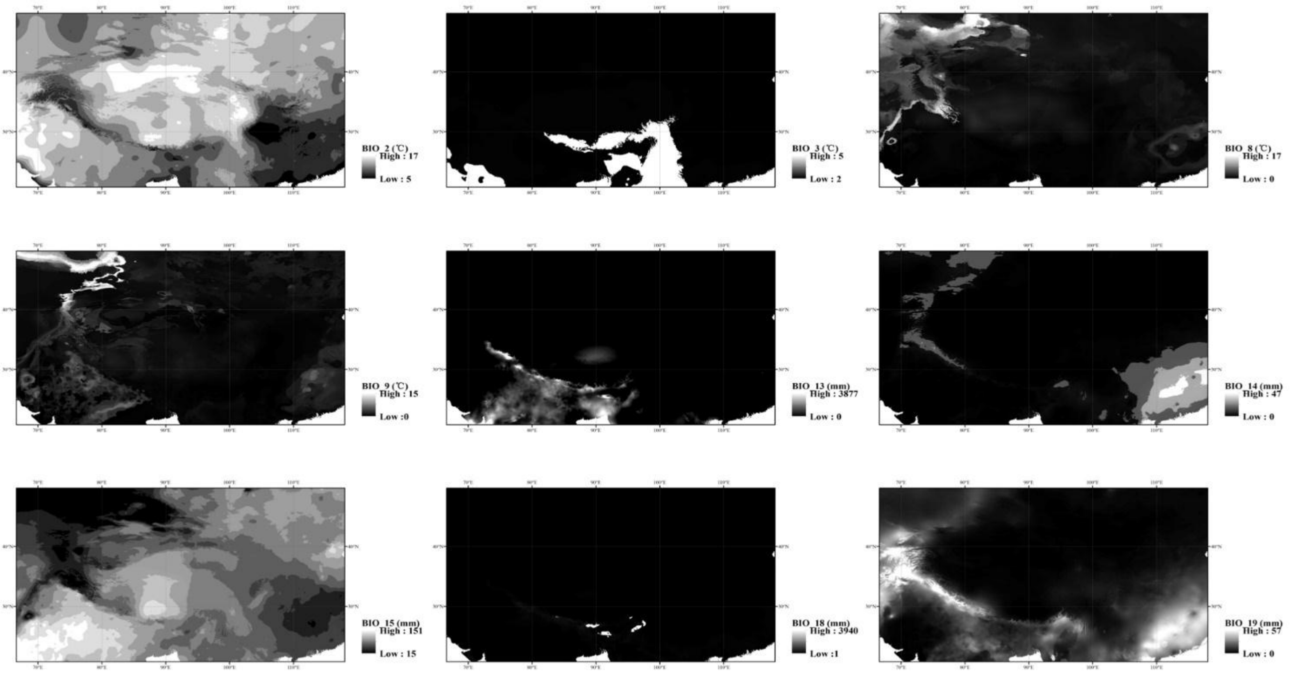

Supplementary Fig. S7. **Standard deviations of multi-GCMs in LGM and 2050 for 9 climate variables used in this study.**

Supplementary Material1 ***Rhodiola*** **records** (Occurrence record data derived from our own field expeditions, from on-line herbarium databases (Global Biodiversity Information Facility Data Portal, GBIF: www.gbif.org; Chinese Virtual Herbarium Data Portal, CVH: www.cvh.org.cn) and from published research[2-11])

species	longitude	latitude
R. alsia	100.16	27.10
R. alsia	91.96	27.99
R. alsia	99.10	28.31
R. alsia	99.00	28.38
R. alsia	85.30	28.55
R. alsia	99.82	28.57
R. alsia	92.50	28.58
R. alsia	99.85	28.59
R. alsia	93.95	29.00
R. alsia	92.35	29.02
R. alsia	93.95	29.05
R. alsia	93.98	29.05
R. alsia	100.02	29.13
R. alsia	100.94	29.55
R. alsia	95.00	29.58
R. alsia	94.58	29.62
R. alsia	94.68	29.62
R. alsia	94.72	29.68
R. alsia	95.69	29.74
R. alsia	92.28	29.87
R. alsia	94.87	29.90
R. alsia	102.28	29.91
R. alsia	101.33	30.05
R. alsia	101.97	30.06
R. alsia	101.40	30.07
R. alsia	101.80	30.07
R. alsia	101.81	30.08
R. alsia	96.88	30.09
R. alsia	100.67	30.15
R. alsia	101.82	30.15
R. alsia	102.32	30.21
R. alsia	99.55	30.33
R. alsia	102.82	30.52
R. alsia	101.27	31.02
R. alsia	102.14	31.16
R. alsia	97.33	31.28
R. alsia	95.63	31.45
R. alsia	100.76	31.57
R. alsia	101.45	31.61
R. alsia	98.82	31.65
R. alsia	103.93	31.65
R. alsia	94.92	31.68
R. alsia	102.67	31.85
R. alsia	102.67	31.87
R. alsia	98.90	31.93

R. alsia	102.65	31.95
R. alsia	100.22	32.05
R. alsia	102.38	32.44
R. alsia	103.60	32.64
R. alsia	104.33	32.74
R. alsia	97.01	33.02
R. alsia	97.55	33.13
R. alsia	97.43	33.20
R. alsia	100.38	33.28
R. alsia	100.27	33.40
R. alsia	103.61	33.64
R. alsia	99.70	33.79
R. alsia	97.62	34.08
R. alsia	97.97	34.28
R. alsia	98.12	34.45
R. alsia	99.67	34.57
R. alsia	100.82	34.71
R. alsia	98.47	34.87
R. alsia	99.69	35.24
R. alsia	94.56	35.88
R. alsia	101.70	36.49
R. alsia	100.47	36.55
R. alsia	99.51	37.04
R. alsia	101.81	37.05
R. alsia	101.41	37.25
R. alsia	102.86	37.26
R. bupleuroides	102.91	26.24
R. bupleuroides	98.97	26.42
R. bupleuroides	99.02	26.59
R. bupleuroides	100.23	26.88
R. bupleuroides	99.05	27.03
R. bupleuroides	100.24	27.04
R. bupleuroides	100.22	27.11
R. bupleuroides	100.67	27.18
R. bupleuroides	98.69	27.20
R. bupleuroides	98.70	27.21
R. bupleuroides	98.72	27.21
R. bupleuroides	98.71	27.22
R. bupleuroides	100.13	27.37
R. bupleuroides	88.91	27.39
R. bupleuroides	88.93	27.42
R. bupleuroides	99.88	27.48
R. bupleuroides	88.98	27.51
R. bupleuroides	88.88	27.52
R. bupleuroides	100.67	27.55
R. bupleuroides	98.96	27.58
R. bupleuroides	99.78	27.60
R. bupleuroides	98.91	27.61
R. bupleuroides	99.81	27.62

R. bupleuroides	99.14	27.68
R. bupleuroides	88.75	27.72
R. bupleuroides	89.16	27.72
R. bupleuroides	100.05	27.74
R. bupleuroides	99.59	27.80
R. bupleuroides	86.77	27.83
R. bupleuroides	99.99	27.83
R. bupleuroides	91.79	27.88
R. bupleuroides	86.80	27.92
R. bupleuroides	88.59	27.93
R. bupleuroides	85.98	27.94
R. bupleuroides	86.61	27.94
R. bupleuroides	91.86	27.95
R. bupleuroides	87.13	27.98
R. bupleuroides	87.67	27.98
R. bupleuroides	99.62	27.98
R. bupleuroides	85.88	27.99
R. bupleuroides	85.99	27.99
R. bupleuroides	98.48	27.99
R. bupleuroides	87.68	28.01
R. bupleuroides	85.99	28.02
R. bupleuroides	87.68	28.02
R. bupleuroides	86.00	28.03
R. bupleuroides	85.44	28.04
R. bupleuroides	91.94	28.06
R. bupleuroides	101.31	28.06
R. bupleuroides	92.36	28.07
R. bupleuroides	89.04	28.11
R. bupleuroides	99.83	28.13
R. bupleuroides	86.85	28.15
R. bupleuroides	99.94	28.16
R. bupleuroides	86.84	28.17
R. bupleuroides	86.84	28.18
R. bupleuroides	87.19	28.18
R. bupleuroides	91.96	28.18
R. bupleuroides	86.82	28.20
R. bupleuroides	87.18	28.20
R. bupleuroides	85.57	28.22
R. bupleuroides	92.27	28.24
R. bupleuroides	86.82	28.25
R. bupleuroides	86.81	28.27
R. bupleuroides	86.82	28.29
R. bupleuroides	86.83	28.30
R. bupleuroides	99.15	28.31
R. bupleuroides	92.91	28.34
R. bupleuroides	98.99	28.34
R. bupleuroides	99.05	28.35
R. bupleuroides	85.36	28.39
R. bupleuroides	87.74	28.40

R. bupleuroides	98.96	28.40
R. bupleuroides	98.96	28.41
R. bupleuroides	98.94	28.42
R. bupleuroides	85.30	28.43
R. bupleuroides	92.89	28.53
R. bupleuroides	98.91	28.54
R. bupleuroides	99.85	28.59
R. bupleuroides	99.84	28.60
R. bupleuroides	98.90	28.61
R. bupleuroides	84.98	28.65
R. bupleuroides	87.19	28.65
R. bupleuroides	98.94	28.66
R. bupleuroides	83.98	28.68
R. bupleuroides	98.78	28.69
R. bupleuroides	83.67	28.72
R. bupleuroides	99.20	28.73
R. bupleuroides	97.37	28.74
R. bupleuroides	99.22	28.82
R. bupleuroides	85.42	28.90
R. bupleuroides	100.28	28.92
R. bupleuroides	83.78	28.93
R. bupleuroides	93.23	28.93
R. bupleuroides	99.76	28.93
R. bupleuroides	91.09	28.94
R. bupleuroides	99.75	28.95
R. bupleuroides	93.90	29.03
R. bupleuroides	87.53	29.04
R. bupleuroides	100.30	29.04
R. bupleuroides	87.56	29.07
R. bupleuroides	97.28	29.08
R. bupleuroides	100.25	29.08
R. bupleuroides	93.44	29.09
R. bupleuroides	100.00	29.12
R. bupleuroides	100.04	29.13
R. bupleuroides	97.21	29.17
R. bupleuroides	90.63	29.21
R. bupleuroides	100.14	29.21
R. bupleuroides	94.28	29.23
R. bupleuroides	97.10	29.23
R. bupleuroides	88.88	29.26
R. bupleuroides	100.08	29.27
R. bupleuroides	92.12	29.28
R. bupleuroides	91.74	29.30
R. bupleuroides	91.98	29.30
R. bupleuroides	88.92	29.31
R. bupleuroides	89.71	29.32
R. bupleuroides	98.13	29.36
R. bupleuroides	96.71	29.47
R. bupleuroides	86.40	29.48

R. bupleuroides	89.48	29.49
R. bupleuroides	94.92	29.58
R. bupleuroides	98.30	29.58
R. bupleuroides	81.00	29.61
R. bupleuroides	89.14	29.65
R. bupleuroides	91.14	29.65
R. bupleuroides	94.35	29.67
R. bupleuroides	98.52	29.68
R. bupleuroides	91.03	29.72
R. bupleuroides	98.45	29.72
R. bupleuroides	97.76	29.73
R. bupleuroides	91.90	29.77
R. bupleuroides	91.78	29.79
R. bupleuroides	91.57	29.81
R. bupleuroides	92.34	29.83
R. bupleuroides	84.18	29.88
R. bupleuroides	92.52	29.88
R. bupleuroides	93.26	29.88
R. bupleuroides	93.66	29.89
R. bupleuroides	93.16	29.93
R. bupleuroides	93.10	29.97
R. bupleuroides	102.01	29.99
R. bupleuroides	93.08	30.00
R. bupleuroides	100.86	30.00
R. bupleuroides	102.08	30.01
R. bupleuroides	84.00	30.02
R. bupleuroides	93.91	30.02
R. bupleuroides	100.89	30.04
R. bupleuroides	84.19	30.07
R. bupleuroides	91.78	30.09
R. bupleuroides	102.01	30.09
R. bupleuroides	80.83	30.10
R. bupleuroides	101.48	30.10
R. bupleuroides	92.13	30.12
R. bupleuroides	90.46	30.14
R. bupleuroides	101.82	30.15
R. bupleuroides	83.40	30.17
R. bupleuroides	99.99	30.18
R. bupleuroides	101.75	30.18
R. bupleuroides	80.28	30.25
R. bupleuroides	99.42	30.25
R. bupleuroides	99.28	30.30
R. bupleuroides	101.52	30.31
R. bupleuroides	99.55	30.33
R. bupleuroides	101.99	30.34
R. bupleuroides	79.83	30.42
R. bupleuroides	99.52	30.45
R. bupleuroides	99.50	30.47
R. bupleuroides	91.11	30.55

R. bupleuroides	91.26	30.59
R. bupleuroides	79.60	30.68
R. bupleuroides	91.50	30.72
R. bupleuroides	81.61	30.81
R. bupleuroides	100.69	30.82
R. bupleuroides	81.30	30.99
R. bupleuroides	82.45	31.04
R. bupleuroides	93.11	31.07
R. bupleuroides	97.52	31.29
R. bupleuroides	97.99	31.29
R. bupleuroides	97.52	31.38
R. bupleuroides	92.98	31.40
R. bupleuroides	93.70	31.48
R. bupleuroides	98.22	31.49
R. bupleuroides	98.20	31.51
R. bupleuroides	98.57	31.59
R. bupleuroides	85.17	31.62
R. bupleuroides	94.55	31.63
R. bupleuroides	85.03	31.72
R. bupleuroides	99.73	31.83
R. bupleuroides	100.73	31.88
R. bupleuroides	98.81	32.03
R. bupleuroides	77.13	32.17
R. bupleuroides	77.00	33.00
R. bupleuroides	76.28	33.02
R. bupleuroides	76.35	33.13
R. bupleuroides	75.33	34.00
R. bupleuroides	75.33	34.03
R. bupleuroides	75.23	34.15
R. bupleuroides	74.65	34.50
R. chryanthemifolia	100.09	25.68
R. chryanthemifolia	101.56	27.36
R. chryanthemifolia	98.38	27.73
R. chryanthemifolia	99.12	27.73
R. chryanthemifolia	89.00	27.75
R. chryanthemifolia	86.73	27.77
R. chryanthemifolia	99.60	27.80
R. chryanthemifolia	101.58	27.86
R. chryanthemifolia	98.96	28.07
R. chryanthemifolia	99.32	28.27
R. chryanthemifolia	99.17	28.30
R. chryanthemifolia	99.08	28.34
R. chryanthemifolia	99.11	28.34
R. chryanthemifolia	99.76	28.37
R. chryanthemifolia	99.01	28.42
R. chryanthemifolia	98.43	28.44
R. chryanthemifolia	98.88	28.45
R. chryanthemifolia	101.22	28.48
R. chryanthemifolia	98.48	28.50

R. chryanthemifolia	100.99	28.57
R. chryanthemifolia	98.63	28.69
R. chryanthemifolia	98.66	28.70
R. chryanthemifolia	99.87	28.77
R. chryanthemifolia	99.31	28.80
R. chryanthemifolia	99.78	28.93
R. chryanthemifolia	99.78	28.94
R. chryanthemifolia	90.47	28.98
R. chryanthemifolia	93.23	29.00
R. chryanthemifolia	99.75	29.01
R. chryanthemifolia	92.30	29.04
R. chryanthemifolia	87.56	29.07
R. chryanthemifolia	93.08	29.08
R. chryanthemifolia	99.63	29.10
R. chryanthemifolia	90.41	29.11
R. chryanthemifolia	92.52	29.12
R. chryanthemifolia	100.00	29.12
R. chryanthemifolia	101.40	29.14
R. chryanthemifolia	90.47	29.16
R. chryanthemifolia	90.51	29.16
R. chryanthemifolia	94.23	29.21
R. chryanthemifolia	92.09	29.24
R. chryanthemifolia	92.28	29.24
R. chryanthemifolia	90.62	29.25
R. chryanthemifolia	91.97	29.29
R. chryanthemifolia	90.72	29.30
R. chryanthemifolia	90.28	29.33
R. chryanthemifolia	90.88	29.35
R. chryanthemifolia	99.43	29.37
R. chryanthemifolia	99.42	29.39
R. chryanthemifolia	88.90	29.40
R. chryanthemifolia	88.86	29.43
R. chryanthemifolia	94.91	29.54
R. chryanthemifolia	91.05	29.65
R. chryanthemifolia	91.12	29.66
R. chryanthemifolia	101.90	29.66
R. chryanthemifolia	91.03	29.67
R. chryanthemifolia	91.05	29.67
R. chryanthemifolia	94.38	29.67
R. chryanthemifolia	94.40	29.67
R. chryanthemifolia	91.38	29.68
R. chryanthemifolia	93.26	29.88
R. chryanthemifolia	93.30	29.88
R. chryanthemifolia	92.85	29.97
R. chryanthemifolia	100.36	29.99
R. chryanthemifolia	101.31	30.04
R. chryanthemifolia	97.63	30.58
R. chryanthemifolia	97.26	30.69
R. chryanthemifolia	101.46	30.72

R. chryanthemifolia	97.26	31.16
R. chryanthemifolia	94.53	31.80
R. chryanthemifolia	103.90	33.15
R. crenulata	100.18	27.03
R. crenulata	100.19	27.06
R. crenulata	100.18	27.12
R. crenulata	100.16	27.18
R. crenulata	100.09	27.32
R. crenulata	99.76	27.69
R. crenulata	89.21	27.78
R. crenulata	88.70	27.83
R. crenulata	86.82	27.91
R. crenulata	86.84	27.91
R. crenulata	99.91	27.96
R. crenulata	88.77	27.97
R. crenulata	99.97	27.97
R. crenulata	87.05	27.98
R. crenulata	87.70	27.99
R. crenulata	87.03	28.00
R. crenulata	87.70	28.01
R. crenulata	99.90	28.11
R. crenulata	99.89	28.12
R. crenulata	86.01	28.16
R. crenulata	86.81	28.18
R. crenulata	86.77	28.19
R. crenulata	86.82	28.20
R. crenulata	85.42	28.24
R. crenulata	99.03	28.33
R. crenulata	92.47	28.46
R. crenulata	98.73	28.48
R. crenulata	90.55	28.50
R. crenulata	98.80	28.51
R. crenulata	85.76	28.56
R. crenulata	100.99	28.58
R. crenulata	99.85	28.59
R. crenulata	98.63	28.65
R. crenulata	97.43	28.70
R. crenulata	97.37	28.78
R. crenulata	99.74	28.82
R. crenulata	93.11	28.93
R. crenulata	97.49	28.96
R. crenulata	98.66	28.96
R. crenulata	87.64	29.08
R. crenulata	92.42	29.10
R. crenulata	100.04	29.13
R. crenulata	100.04	29.15
R. crenulata	101.44	29.15
R. crenulata	97.22	29.17
R. crenulata	98.98	29.24

R. crenulata	95.56	29.35
R. crenulata	101.98	29.51
R. crenulata	94.22	29.53
R. crenulata	90.76	29.58
R. crenulata	94.50	29.62
R. crenulata	94.68	29.62
R. crenulata	94.70	29.62
R. crenulata	94.41	29.64
R. crenulata	94.70	29.64
R. crenulata	101.98	29.67
R. crenulata	98.00	29.71
R. crenulata	92.31	29.79
R. crenulata	92.36	29.86
R. crenulata	92.32	29.92
R. crenulata	89.09	30.07
R. crenulata	101.82	30.10
R. crenulata	90.42	30.20
R. crenulata	80.25	30.30
R. crenulata	80.62	30.33
R. crenulata	101.76	30.41
R. crenulata	79.83	30.42
R. crenulata	84.01	30.42
R. crenulata	101.83	30.42
R. crenulata	81.29	30.50
R. crenulata	102.15	30.53
R. crenulata	93.49	30.59
R. crenulata	79.60	30.68
R. crenulata	81.64	30.87
R. crenulata	96.94	31.08
R. crenulata	99.66	31.08
R. crenulata	80.93	31.23
R. crenulata	98.57	31.81
R. crenulata	98.91	31.94
R. crenulata	94.13	31.95
R. crenulata	98.91	31.95
R. crenulata	102.22	31.95
R. crenulata	98.80	32.03
R. crenulata	95.62	32.28
R. crenulata	77.25	32.37
R. crenulata	93.73	32.46
R. crenulata	100.54	32.58
R. crenulata	98.05	32.66
R. crenulata	77.50	32.70
R. crenulata	97.92	32.78
R. crenulata	97.12	32.87
R. crenulata	98.07	32.98
R. crenulata	97.01	33.02
R. crenulata	77.59	33.03
R. crenulata	99.56	33.36

R. crenulata	99.33	34.32
R. dumulosa	100.14	26.99
R. dumulosa	91.90	28.12
R. dumulosa	86.84	28.16
R. dumulosa	98.79	28.36
R. dumulosa	90.58	28.43
R. dumulosa	92.23	28.47
R. dumulosa	90.63	28.50
R. dumulosa	85.40	28.93
R. dumulosa	99.80	28.93
R. dumulosa	93.98	29.05
R. dumulosa	92.28	29.87
R. dumulosa	102.23	29.91
R. dumulosa	101.96	30.06
R. dumulosa	90.58	30.32
R. dumulosa	90.55	30.35
R. dumulosa	110.26	31.45
R. dumulosa	95.33	31.50
R. dumulosa	94.92	31.68
R. dumulosa	94.52	31.70
R. dumulosa	102.66	31.84
R. dumulosa	98.92	31.93
R. dumulosa	97.07	32.08
R. dumulosa	97.27	32.09
R. dumulosa	97.27	32.12
R. dumulosa	97.05	32.15
R. dumulosa	103.83	32.59
R. dumulosa	97.01	33.02
R. dumulosa	101.13	33.43
R. dumulosa	91.20	33.47
R. dumulosa	107.60	33.72
R. dumulosa	107.77	33.95
R. dumulosa	107.79	33.96
R. dumulosa	107.25	33.98
R. dumulosa	107.81	34.00
R. dumulosa	103.75	34.93
R. dumulosa	106.05	35.56
R. dumulosa	103.20	35.59
R. dumulosa	102.48	35.68
R. dumulosa	103.95	35.75
R. dumulosa	104.11	35.84
R. dumulosa	94.57	35.87
R. dumulosa	101.79	35.87
R. dumulosa	103.76	36.08
R. dumulosa	102.72	36.11
R. dumulosa	102.72	36.12
R. dumulosa	102.44	36.51
R. dumulosa	102.39	36.66
R. dumulosa	102.38	36.67

R. dumulosa	102.37	36.72
R. dumulosa	103.26	36.74
R. dumulosa	102.53	36.77
R. dumulosa	102.28	36.79
R. dumulosa	102.66	36.79
R. dumulosa	102.48	36.82
R. dumulosa	102.27	36.84
R. dumulosa	102.15	36.85
R. dumulosa	101.70	36.93
R. dumulosa	103.14	36.97
R. dumulosa	101.76	37.03
R. dumulosa	101.57	37.14
R. dumulosa	101.57	37.16
R. dumulosa	101.91	37.16
R. dumulosa	102.67	37.23
R. dumulosa	101.54	37.24
R. dumulosa	102.01	37.28
R. dumulosa	102.01	37.29
R. dumulosa	101.76	37.50
R. dumulosa	111.49	37.90
R. dumulosa	111.52	37.90
R. dumulosa	100.25	38.13
R. dumulosa	99.67	38.63
R. dumulosa	111.84	38.72
R. dumulosa	111.87	38.73
R. dumulosa	111.89	38.73
R. dumulosa	111.93	38.73
R. dumulosa	111.93	38.74
R. dumulosa	111.93	38.75
R. dumulosa	105.94	38.84
R. dumulosa	105.69	38.85
R. dumulosa	105.94	38.85
R. dumulosa	113.55	39.00
R. dumulosa	113.60	39.01
R. dumulosa	113.49	39.04
R. dumulosa	113.53	39.05
R. dumulosa	113.57	39.08
R. dumulosa	114.97	39.91
R. dumulosa	115.05	39.94
R. dumulosa	115.06	39.95
R. dumulosa	115.49	40.01
R. dumulosa	115.48	40.03
R. dumulosa	115.50	40.03
R. dumulosa	115.47	40.04
R. dumulosa	111.26	40.84
R. fastigiata	100.11	25.65
R. fastigiata	100.10	25.66
R. fastigiata	100.09	25.67
R. fastigiata	102.91	26.15

R. fastigiata	102.96	26.15
R. fastigiata	102.85	26.16
R. fastigiata	100.24	26.89
R. fastigiata	99.67	26.91
R. fastigiata	100.21	27.09
R. fastigiata	100.17	27.13
R. fastigiata	100.17	27.15
R. fastigiata	103.06	27.17
R. fastigiata	102.43	27.27
R. fastigiata	98.81	27.75
R. fastigiata	99.58	27.77
R. fastigiata	98.75	27.79
R. fastigiata	99.60	27.79
R. fastigiata	98.75	27.81
R. fastigiata	99.71	27.82
R. fastigiata	99.72	27.83
R. fastigiata	99.52	27.88
R. fastigiata	101.60	27.88
R. fastigiata	86.71	27.91
R. fastigiata	99.70	27.91
R. fastigiata	101.30	27.93
R. fastigiata	87.05	27.98
R. fastigiata	101.17	27.99
R. fastigiata	101.34	27.99
R. fastigiata	101.34	28.01
R. fastigiata	92.00	28.03
R. fastigiata	85.99	28.07
R. fastigiata	98.60	28.10
R. fastigiata	85.95	28.14
R. fastigiata	99.81	28.15
R. fastigiata	85.92	28.16
R. fastigiata	86.85	28.16
R. fastigiata	103.22	28.27
R. fastigiata	98.75	28.31
R. fastigiata	87.05	28.33
R. fastigiata	99.11	28.36
R. fastigiata	98.65	28.39
R. fastigiata	98.77	28.40
R. fastigiata	92.05	28.41
R. fastigiata	99.08	28.43
R. fastigiata	98.74	28.44
R. fastigiata	99.06	28.45
R. fastigiata	103.22	28.46
R. fastigiata	98.89	28.49
R. fastigiata	98.95	28.49
R. fastigiata	98.91	28.54
R. fastigiata	99.86	28.59
R. fastigiata	87.19	28.60
R. fastigiata	99.84	28.60

R. fastigiata	98.62	28.65
R. fastigiata	98.66	28.65
R. fastigiata	83.98	28.68
R. fastigiata	102.40	28.70
R. fastigiata	97.36	28.71
R. fastigiata	99.87	28.73
R. fastigiata	102.37	28.74
R. fastigiata	97.55	28.79
R. fastigiata	93.07	28.91
R. fastigiata	101.51	29.00
R. fastigiata	92.35	29.04
R. fastigiata	100.30	29.04
R. fastigiata	92.32	29.05
R. fastigiata	100.89	29.07
R. fastigiata	102.19	29.07
R. fastigiata	93.42	29.09
R. fastigiata	100.02	29.10
R. fastigiata	99.94	29.14
R. fastigiata	92.38	29.17
R. fastigiata	94.25	29.19
R. fastigiata	94.23	29.21
R. fastigiata	94.16	29.24
R. fastigiata	95.75	29.33
R. fastigiata	95.55	29.36
R. fastigiata	95.52	29.39
R. fastigiata	95.71	29.39
R. fastigiata	100.98	29.40
R. fastigiata	96.86	29.42
R. fastigiata	94.86	29.46
R. fastigiata	94.85	29.49
R. fastigiata	97.68	29.53
R. fastigiata	97.48	29.54
R. fastigiata	94.59	29.57
R. fastigiata	94.59	29.59
R. fastigiata	100.90	29.59
R. fastigiata	94.63	29.60
R. fastigiata	94.65	29.61
R. fastigiata	94.71	29.61
R. fastigiata	94.65	29.63
R. fastigiata	94.32	29.64
R. fastigiata	87.53	29.65
R. fastigiata	94.44	29.65
R. fastigiata	94.68	29.65
R. fastigiata	94.69	29.66
R. fastigiata	96.04	29.71
R. fastigiata	91.02	29.72
R. fastigiata	97.75	29.74
R. fastigiata	97.23	29.79
R. fastigiata	94.56	29.80

R. fastigiata	97.16	29.81
R. fastigiata	95.29	29.91
R. fastigiata	85.73	29.93
R. fastigiata	84.33	29.94
R. fastigiata	94.72	29.94
R. fastigiata	95.44	29.96
R. fastigiata	101.99	29.98
R. fastigiata	101.97	29.99
R. fastigiata	102.01	29.99
R. fastigiata	100.27	30.00
R. fastigiata	100.87	30.02
R. fastigiata	101.96	30.06
R. fastigiata	101.80	30.11
R. fastigiata	101.87	30.14
R. fastigiata	101.86	30.15
R. fastigiata	102.52	30.16
R. fastigiata	101.76	30.17
R. fastigiata	81.29	30.20
R. fastigiata	99.56	30.26
R. fastigiata	99.54	30.29
R. fastigiata	102.39	30.40
R. fastigiata	97.60	30.44
R. fastigiata	97.65	30.44
R. fastigiata	97.61	30.46
R. fastigiata	102.80	30.47
R. fastigiata	81.29	30.51
R. fastigiata	97.27	30.69
R. fastigiata	100.37	30.75
R. fastigiata	102.98	31.10
R. fastigiata	102.51	31.13
R. fastigiata	102.14	31.33
R. fastigiata	93.75	31.39
R. fastigiata	85.56	31.47
R. fastigiata	102.20	31.48
R. fastigiata	95.29	31.53
R. fastigiata	102.27	31.54
R. fastigiata	95.43	31.57
R. fastigiata	101.69	31.79
R. fastigiata	101.80	31.81
R. fastigiata	79.92	31.84
R. fastigiata	102.19	31.87
R. fastigiata	102.19	31.89
R. fastigiata	102.22	31.90
R. fastigiata	84.73	31.92
R. fastigiata	101.02	31.94
R. fastigiata	98.80	32.02
R. fastigiata	102.65	32.04
R. fastigiata	102.78	32.13
R. fastigiata	103.60	32.66

R. fastigiata	103.75	32.75
R. fastigiata	100.54	32.89
R. fastigiata	100.77	32.96
R. fastigiata	79.62	33.31
R. fastigiata	102.54	35.17
R. fastigiata	93.08	35.22
R. fastigiata	93.09	35.24
R. fastigiata	102.03	37.28
R. fastigiata	97.29	37.75
R. fastigiata	101.26	37.75
R. forrestii	99.91	26.54
R. forrestii	100.18	26.56
R. forrestii	100.14	26.64
R. forrestii	102.71	26.65
R. forrestii	100.23	26.88
R. forrestii	100.19	27.01
R. forrestii	100.18	27.02
R. forrestii	100.18	27.03
R. forrestii	102.75	27.17
R. forrestii	99.29	27.18
R. forrestii	103.07	27.18
R. forrestii	100.10	27.36
R. forrestii	101.56	27.38
R. forrestii	102.79	27.61
R. forrestii	99.12	27.71
R. forrestii	99.81	27.79
R. forrestii	99.63	27.81
R. forrestii	99.71	27.82
R. forrestii	100.00	27.83
R. forrestii	101.61	27.87
R. forrestii	101.25	27.92
R. forrestii	103.39	27.93
R. forrestii	100.72	28.07
R. forrestii	101.21	28.12
R. forrestii	100.82	28.17
R. forrestii	103.52	28.31
R. forrestii	103.37	28.42
R. forrestii	100.99	28.54
R. forrestii	102.38	28.68
R. forrestii	101.48	28.98
R. forrestii	101.49	28.99
R. forrestii	101.50	29.00
R. forrestii	102.91	29.02
R. forrestii	101.77	29.57
R. forrestii	101.77	29.58
R. forrestii	101.99	29.68
R. forrestii	101.30	30.05
R. forrestii	102.01	31.34
R. forrestii	102.81	31.66

R. forrestii	103.87	31.68
R. forrestii	100.95	32.27
R. henryi	103.11	27.82
R. henryi	108.68	27.88
R. henryi	108.69	27.92
R. henryi	108.79	28.56
R. henryi	107.26	28.91
R. henryi	107.16	28.94
R. henryi	107.19	28.98
R. henryi	107.15	29.01
R. henryi	107.19	29.01
R. henryi	107.19	29.03
R. henryi	102.59	29.19
R. henryi	107.43	29.23
R. henryi	107.45	29.46
R. henryi	108.05	29.51
R. henryi	103.33	29.53
R. henryi	107.61	29.54
R. henryi	103.35	29.55
R. henryi	108.69	29.59
R. henryi	109.81	29.68
R. henryi	108.28	29.74
R. henryi	108.27	29.77
R. henryi	110.38	29.83
R. henryi	101.96	29.95
R. henryi	101.94	29.97
R. henryi	94.77	29.98
R. henryi	101.53	30.01
R. henryi	110.55	30.02
R. henryi	110.79	30.05
R. henryi	110.26	30.08
R. henryi	110.67	30.14
R. henryi	101.88	30.26
R. henryi	102.86	30.37
R. henryi	109.07	30.50
R. henryi	109.54	30.67
R. henryi	109.64	30.69
R. henryi	102.17	30.76
R. henryi	101.20	31.03
R. henryi	110.93	31.06
R. henryi	110.96	31.10
R. henryi	110.16	31.29
R. henryi	110.78	31.33
R. henryi	109.92	31.36
R. henryi	109.89	31.42
R. henryi	110.23	31.46
R. henryi	109.84	31.47
R. henryi	109.92	31.47
R. henryi	110.31	31.48

R. henryi	110.43	31.49
R. henryi	109.11	31.51
R. henryi	110.35	31.51
R. henryi	110.48	31.53
R. henryi	110.48	31.58
R. henryi	110.83	31.60
R. henryi	110.43	31.65
R. henryi	110.47	31.66
R. henryi	115.56	31.70
R. henryi	111.08	31.71
R. henryi	110.50	31.72
R. henryi	110.47	31.73
R. henryi	111.12	31.73
R. henryi	110.50	31.79
R. henryi	109.41	31.84
R. henryi	108.43	31.88
R. henryi	108.56	31.91
R. henryi	108.41	31.93
R. henryi	108.78	31.95
R. henryi	109.36	32.02
R. henryi	108.84	32.03
R. henryi	110.73	32.03
R. henryi	108.19	32.10
R. henryi	109.28	32.10
R. henryi	108.94	32.19
R. henryi	108.22	32.20
R. henryi	109.03	32.26
R. henryi	104.89	32.39
R. henryi	104.79	32.51
R. henryi	104.66	32.55
R. henryi	106.86	32.61
R. henryi	107.97	32.61
R. henryi	106.99	32.67
R. henryi	105.08	32.68
R. henryi	106.99	32.70
R. henryi	106.86	32.72
R. henryi	103.82	32.75
R. henryi	105.25	32.79
R. henryi	104.83	32.81
R. henryi	104.44	32.86
R. henryi	104.59	32.90
R. henryi	105.30	32.98
R. henryi	109.42	32.99
R. henryi	108.27	33.16
R. henryi	110.19	33.20
R. henryi	108.63	33.33
R. henryi	108.41	33.35
R. henryi	105.49	33.38
R. henryi	108.50	33.39

R. henryi	108.41	33.40
R. henryi	107.92	33.43
R. henryi	106.81	33.53
R. henryi	108.55	33.55
R. henryi	107.66	33.58
R. henryi	109.00	33.62
R. henryi	108.69	33.66
R. henryi	111.81	33.67
R. henryi	109.02	33.80
R. henryi	107.53	33.81
R. henryi	108.01	33.81
R. henryi	104.08	33.93
R. henryi	111.17	33.93
R. henryi	107.76	33.99
R. henryi	111.16	34.01
R. henryi	107.72	34.02
R. henryi	107.71	34.05
R. henryi	107.72	34.08
R. henryi	109.48	34.08
R. henryi	107.87	34.09
R. henryi	107.88	34.11
R. henryi	107.10	34.13
R. henryi	103.88	34.16
R. henryi	107.18	34.24
R. henryi	106.83	34.27
R. henryi	106.14	34.35
R. henryi	110.08	34.46
R. henryi	110.08	34.49
R. henryi	106.25	34.54
R. himalensis	88.84	27.40
R. himalensis	88.86	27.42
R. himalensis	87.99	27.43
R. himalensis	88.97	27.49
R. himalensis	86.47	27.52
R. himalensis	89.16	27.72
R. himalensis	86.43	27.73
R. himalensis	87.86	27.76
R. himalensis	86.72	27.91
R. himalensis	86.60	27.94
R. himalensis	86.60	27.96
R. himalensis	85.88	28.00
R. himalensis	87.69	28.01
R. himalensis	85.42	28.07
R. himalensis	85.57	28.10
R. himalensis	85.97	28.13
R. himalensis	90.90	28.35
R. himalensis	86.65	28.37
R. himalensis	85.31	28.38
R. himalensis	85.40	28.40

R. himalensis	98.91	28.49
R. himalensis	92.50	28.58
R. himalensis	92.53	28.60
R. himalensis	83.83	28.72
R. himalensis	99.91	28.77
R. himalensis	93.23	28.93
R. himalensis	93.43	29.10
R. himalensis	100.04	29.13
R. himalensis	100.08	29.16
R. himalensis	100.19	29.46
R. himalensis	94.93	29.49
R. himalensis	91.05	29.67
R. himalensis	91.07	29.67
R. himalensis	91.13	29.68
R. himalensis	95.70	29.75
R. himalensis	100.86	30.00
R. himalensis	101.39	30.05
R. himalensis	101.40	30.07
R. himalensis	101.78	30.07
R. himalensis	101.81	30.07
R. himalensis	92.13	30.12
R. himalensis	97.27	30.17
R. himalensis	81.30	30.19
R. himalensis	91.36	30.20
R. himalensis	90.62	30.30
R. himalensis	90.60	30.32
R. himalensis	96.97	31.08
R. himalensis	81.32	31.09
R. himalensis	103.71	31.30
R. himalensis	94.97	31.70
R. himalensis	101.71	31.77
R. himalensis	102.68	31.86
R. himalensis	99.12	31.87
R. himalensis	96.58	32.03
R. himalensis	96.53	32.07
R. himalensis	95.29	32.50
R. himalensis	100.46	32.58
R. himalensis	95.33	32.61
R. himalensis	95.61	32.78
R. himalensis	97.16	32.84
R. himalensis	95.15	32.87
R. himalensis	95.48	32.88
R. himalensis	95.61	32.98
R. himalensis	95.19	33.08
R. himalensis	97.13	33.36
R. himalensis	97.13	33.46
R. himalensis	100.93	33.53
R. himalensis	100.63	33.73
R. himalensis	101.05	33.77

R. himalensis	96.52	33.89
R. himalensis	99.45	34.00
R. himalensis	100.22	34.37
R. himalensis	100.21	34.48
R. himalensis	100.26	34.49
R. himalensis	99.97	34.52
R. himalensis	100.48	34.53
R. himalensis	100.46	34.54
R. himalensis	100.56	34.56
R. himalensis	101.34	34.58
R. himalensis	100.82	34.71
R. himalensis	100.70	34.77
R. himalensis	75.72	34.93
R. himalensis	75.47	34.97
R. himalensis	102.24	35.18
R. himalensis	101.91	35.88
R. himalensis	101.69	36.01
R. himalensis	101.62	36.28
R. himalensis	100.01	36.49
R. himalensis	99.80	36.50
R. himalensis	102.50	37.02
R. himalensis	101.58	37.11
R. himalensis	102.01	37.28
R. kirilowii	100.19	27.03
R. kirilowii	100.16	27.05
R. kirilowii	100.19	27.05
R. kirilowii	100.20	27.11
R. kirilowii	99.12	27.71
R. kirilowii	99.56	27.88
R. kirilowii	98.47	27.98
R. kirilowii	98.46	27.99
R. kirilowii	98.50	27.99
R. kirilowii	98.87	28.04
R. kirilowii	98.96	28.07
R. kirilowii	86.00	28.10
R. kirilowii	99.82	28.16
R. kirilowii	103.16	28.24
R. kirilowii	99.07	28.33
R. kirilowii	98.99	28.38
R. kirilowii	99.01	28.38
R. kirilowii	98.99	28.39
R. kirilowii	85.23	28.55
R. kirilowii	97.95	28.59
R. kirilowii	102.39	28.63
R. kirilowii	100.62	28.74
R. kirilowii	93.80	28.77
R. kirilowii	102.10	28.96
R. kirilowii	100.02	29.12
R. kirilowii	99.65	29.14

R. kirilowii	99.93	29.15
R. kirilowii	101.52	29.22
R. kirilowii	100.08	29.27
R. kirilowii	100.08	29.28
R. kirilowii	97.04	29.32
R. kirilowii	91.53	29.37
R. kirilowii	94.92	29.50
R. kirilowii	95.00	29.58
R. kirilowii	98.15	29.58
R. kirilowii	102.09	29.59
R. kirilowii	102.09	29.61
R. kirilowii	94.58	29.62
R. kirilowii	94.68	29.62
R. kirilowii	94.32	29.64
R. kirilowii	102.63	29.67
R. kirilowii	95.70	29.79
R. kirilowii	95.78	29.87
R. kirilowii	100.87	30.00
R. kirilowii	101.87	30.00
R. kirilowii	101.84	30.04
R. kirilowii	101.33	30.07
R. kirilowii	91.25	30.12
R. kirilowii	92.12	30.12
R. kirilowii	97.32	30.16
R. kirilowii	84.07	30.18
R. kirilowii	101.86	30.26
R. kirilowii	101.86	30.27
R. kirilowii	101.52	30.31
R. kirilowii	94.78	30.36
R. kirilowii	102.53	30.47
R. kirilowii	102.93	30.92
R. kirilowii	101.24	31.02
R. kirilowii	96.95	31.08
R. kirilowii	96.97	31.08
R. kirilowii	96.87	31.11
R. kirilowii	96.60	31.18
R. kirilowii	102.14	31.29
R. kirilowii	103.62	31.37
R. kirilowii	102.08	31.42
R. kirilowii	99.99	31.50
R. kirilowii	96.35	31.52
R. kirilowii	103.91	31.66
R. kirilowii	100.76	31.76
R. kirilowii	98.58	31.81
R. kirilowii	101.34	31.86
R. kirilowii	99.12	31.87
R. kirilowii	102.65	31.90
R. kirilowii	102.27	31.91
R. kirilowii	98.90	31.95

R. kirilowii	102.65	31.95
R. kirilowii	102.64	32.06
R. kirilowii	97.07	32.08
R. kirilowii	98.86	32.12
R. kirilowii	102.79	32.13
R. kirilowii	102.58	32.23
R. kirilowii	96.27	32.28
R. kirilowii	96.54	32.30
R. kirilowii	100.46	32.58
R. kirilowii	103.58	32.67
R. kirilowii	100.93	32.71
R. kirilowii	97.07	32.81
R. kirilowii	100.83	32.84
R. kirilowii	97.15	32.85
R. kirilowii	100.67	32.90
R. kirilowii	97.04	32.92
R. kirilowii	103.35	32.96
R. kirilowii	96.86	33.05
R. kirilowii	97.09	33.34
R. kirilowii	100.88	33.52
R. kirilowii	103.51	33.65
R. kirilowii	99.64	33.86
R. kirilowii	99.68	33.90
R. kirilowii	102.90	33.90
R. kirilowii	104.27	33.91
R. kirilowii	107.77	33.95
R. kirilowii	107.78	33.97
R. kirilowii	107.79	33.99
R. kirilowii	107.86	34.02
R. kirilowii	103.91	34.11
R. kirilowii	98.40	34.18
R. kirilowii	100.22	34.37
R. kirilowii	101.52	34.37
R. kirilowii	104.04	34.46
R. kirilowii	104.04	34.47
R. kirilowii	100.56	34.56
R. kirilowii	101.33	34.56
R. kirilowii	100.61	34.65
R. kirilowii	100.63	34.66
R. kirilowii	100.80	34.70
R. kirilowii	100.72	34.73
R. kirilowii	103.31	34.81
R. kirilowii	97.44	34.87
R. kirilowii	103.75	34.94
R. kirilowii	103.75	34.95
R. kirilowii	99.25	34.98
R. kirilowii	100.34	35.05
R. kirilowii	101.82	35.06
R. kirilowii	102.25	35.19

R. kirilowii	102.59	35.19
R. kirilowii	101.90	35.26
R. kirilowii	101.89	35.27
R. kirilowii	102.19	35.55
R. kirilowii	102.33	35.74
R. kirilowii	102.72	36.11
R. kirilowii	102.19	36.29
R. kirilowii	102.23	36.29
R. kirilowii	100.25	36.47
R. kirilowii	100.26	36.51
R. kirilowii	112.04	36.69
R. kirilowii	102.53	36.82
R. kirilowii	112.07	36.85
R. kirilowii	101.32	36.92
R. kirilowii	101.85	37.02
R. kirilowii	101.82	37.03
R. kirilowii	101.57	37.11
R. kirilowii	102.22	37.16
R. kirilowii	102.86	37.26
R. kirilowii	102.01	37.28
R. kirilowii	101.56	37.51
R. kirilowii	111.43	37.87
R. kirilowii	111.49	37.90
R. kirilowii	113.83	38.84
R. kirilowii	113.60	39.01
R. kirilowii	113.57	39.02
R. kirilowii	113.52	39.03
R. kirilowii	113.65	39.06
R. kirilowii	113.58	39.07
R. kirilowii	114.57	39.58
R. kirilowii	114.99	39.91
R. kirilowii	115.49	40.01
R. kirilowii	115.34	40.03
R. kirilowii	115.45	40.03
R. kirilowii	117.49	40.60
R. kirilowii	117.48	40.61
R. kirilowii	80.82	42.51
R. kirilowii	77.15	43.05
R. kirilowii	82.33	43.05
R. kirilowii	86.95	43.11
R. kirilowii	87.07	43.12
R. kirilowii	81.14	43.19
R. kirilowii	87.04	43.26
R. kirilowii	87.18	43.26
R. kirilowii	87.01	43.47
R. kirilowii	87.01	43.50
R. kirilowii	90.05	43.58
R. kirilowii	88.02	43.70
R. kirilowii	88.04	43.85

R. kirilowii	88.24	47.97
R. quadrifida	100.09	27.32
R. quadrifida	102.46	27.34
R. quadrifida	88.95	27.53
R. quadrifida	87.70	27.99
R. quadrifida	101.41	27.99
R. quadrifida	87.68	28.02
R. quadrifida	87.54	28.92
R. quadrifida	101.64	29.03
R. quadrifida	91.78	29.12
R. quadrifida	102.07	30.00
R. quadrifida	81.28	30.33
R. quadrifida	101.62	30.42
R. quadrifida	81.64	30.82
R. quadrifida	97.53	31.26
R. quadrifida	96.46	31.93
R. quadrifida	78.01	31.95
R. quadrifida	96.35	32.07
R. quadrifida	96.51	32.29
R. quadrifida	91.70	32.30
R. quadrifida	83.17	32.44
R. quadrifida	97.68	32.54
R. quadrifida	103.83	32.59
R. quadrifida	95.59	32.76
R. quadrifida	97.18	32.90
R. quadrifida	101.07	33.06
R. quadrifida	104.31	33.88
R. quadrifida	95.75	34.02
R. quadrifida	100.25	34.47
R. quadrifida	101.14	34.56
R. quadrifida	92.65	34.58
R. quadrifida	99.45	34.58
R. quadrifida	101.33	34.60
R. quadrifida	99.55	34.68
R. quadrifida	99.03	34.82
R. quadrifida	97.57	34.92
R. quadrifida	100.64	34.92
R. quadrifida	98.88	35.12
R. quadrifida	101.58	35.18
R. quadrifida	101.61	35.21
R. quadrifida	101.91	35.26
R. quadrifida	94.06	35.63
R. quadrifida	99.87	35.82
R. quadrifida	99.93	35.87
R. quadrifida	99.68	35.89
R. quadrifida	96.47	36.04
R. quadrifida	101.64	36.09
R. quadrifida	101.51	36.26
R. quadrifida	101.62	36.28

R. quadrifida	102.18	36.29
R. quadrifida	100.73	36.49
R. quadrifida	77.48	36.83
R. quadrifida	98.17	36.87
R. quadrifida	103.21	37.15
R. quadrifida	101.28	37.17
R. quadrifida	101.29	37.18
R. quadrifida	101.28	37.19
R. quadrifida	101.64	37.20
R. quadrifida	102.72	37.27
R. quadrifida	101.40	37.34
R. quadrifida	97.42	37.46
R. quadrifida	101.62	37.47
R. quadrifida	76.10	37.62
R. quadrifida	75.19	37.69
R. quadrifida	100.25	38.11
R. quadrifida	100.23	38.13
R. quadrifida	95.39	38.16
R. quadrifida	97.94	38.31
R. quadrifida	99.49	38.44
R. quadrifida	99.57	38.50
R. quadrifida	99.87	38.70
R. quadrifida	75.15	38.78
R. quadrifida	96.81	38.81
R. quadrifida	80.59	42.58
R. quadrifida	86.93	43.16
R. quadrifida	86.07	43.49
R. quadrifida	87.72	49.29
R. quadrifida	88.03	49.41
R. quadrifida	88.15	49.44
R. sacra	86.00	28.10
R. sacra	85.96	28.16
R. sacra	99.03	28.35
R. sacra	91.39	28.42
R. sacra	98.90	28.45
R. sacra	87.87	28.55
R. sacra	92.50	28.58
R. sacra	92.53	28.60
R. sacra	85.72	28.67
R. sacra	90.55	28.83
R. sacra	91.90	28.86
R. sacra	88.00	28.92
R. sacra	93.06	28.93
R. sacra	89.63	28.95
R. sacra	93.39	28.96
R. sacra	90.30	28.97
R. sacra	90.45	28.97
R. sacra	90.45	28.98
R. sacra	90.53	28.98

R. sacra	92.36	29.03
R. sacra	87.59	29.05
R. sacra	87.57	29.07
R. sacra	90.44	29.10
R. sacra	92.57	29.13
R. sacra	94.27	29.21
R. sacra	90.62	29.22
R. sacra	88.88	29.25
R. sacra	88.87	29.27
R. sacra	88.15	29.28
R. sacra	92.12	29.28
R. sacra	91.98	29.30
R. sacra	89.62	29.32
R. sacra	89.97	29.33
R. sacra	90.05	29.33
R. sacra	85.25	29.35
R. sacra	86.15	29.47
R. sacra	90.92	29.53
R. sacra	85.25	29.57
R. sacra	94.53	29.57
R. sacra	91.15	29.58
R. sacra	91.10	29.65
R. sacra	91.12	29.65
R. sacra	91.05	29.67
R. sacra	91.07	29.67
R. sacra	91.45	29.68
R. sacra	98.58	29.68
R. sacra	91.13	29.69
R. sacra	91.12	29.72
R. sacra	90.98	29.73
R. sacra	91.12	29.74
R. sacra	91.22	29.83
R. sacra	91.24	29.84
R. sacra	99.11	29.93
R. sacra	97.46	29.95
R. sacra	93.89	30.00
R. sacra	100.28	30.02
R. sacra	90.56	30.10
R. sacra	91.52	30.28
R. sacra	91.52	30.30
R. sacra	90.90	30.37
R. sacra	98.27	30.86
R. sacra	98.41	31.59
R. sacra	98.57	31.73
R. sacra	90.00	32.00
R. sacra	97.02	32.08
R. sacra	97.01	32.09
R. sacra	97.03	32.12
R. sacra	96.89	32.26

R. sacra	96.85	32.30
R. wallichiana	100.12	27.36
R. wallichiana	88.90	27.53
R. wallichiana	98.58	27.62
R. wallichiana	98.59	27.63
R. wallichiana	98.75	27.77
R. wallichiana	86.80	27.86
R. wallichiana	98.48	27.98
R. wallichiana	98.48	27.99
R. wallichiana	98.97	28.09
R. wallichiana	86.00	28.10
R. wallichiana	85.99	28.12
R. wallichiana	85.57	28.22
R. wallichiana	98.79	28.36
R. wallichiana	99.81	28.57
R. wallichiana	83.62	28.63
R. wallichiana	84.45	28.63
R. wallichiana	83.88	28.82
R. wallichiana	83.78	28.93
R. wallichiana	101.21	29.12
R. wallichiana	83.97	29.22
R. wallichiana	95.50	29.41
R. wallichiana	94.59	29.55
R. wallichiana	94.61	29.65
R. wallichiana	94.87	29.90
R. wallichiana	95.48	29.93
R. wallichiana	81.33	30.13
R. wallichiana	101.57	30.59
R. wallichiana	79.60	30.68
R. wallichiana	101.23	31.01
R. wallichiana	103.14	31.02
R. wallichiana	98.86	31.95
R. wallichiana	100.31	31.97
R. wallichiana	98.87	32.12
R. wallichiana	77.00	33.00
R. wallichiana	107.38	33.97
R. wallichiana	102.22	35.58
R. wallichiana	74.70	36.34
R. wallichiana	100.09	36.57
R. wallichiana	99.97	36.60
R. yunnanensis	100.13	25.63
R. yunnanensis	100.10	25.64
R. yunnanensis	100.12	25.66
R. yunnanensis	100.14	25.69
R. yunnanensis	100.05	25.70
R. yunnanensis	100.13	25.70
R. yunnanensis	100.04	25.73
R. yunnanensis	100.12	25.73
R. yunnanensis	100.10	25.74

R. yunnanensis	100.08	25.75
R. yunnanensis	100.35	25.97
R. yunnanensis	100.37	25.97
R. yunnanensis	102.80	26.01
R. yunnanensis	102.90	26.15
R. yunnanensis	100.71	26.21
R. yunnanensis	99.06	26.47
R. yunnanensis	98.70	26.50
R. yunnanensis	100.14	26.55
R. yunnanensis	98.80	26.58
R. yunnanensis	104.13	26.74
R. yunnanensis	98.94	26.90
R. yunnanensis	101.84	27.01
R. yunnanensis	100.18	27.02
R. yunnanensis	100.10	27.06
R. yunnanensis	99.10	27.08
R. yunnanensis	100.23	27.08
R. yunnanensis	100.25	27.11
R. yunnanensis	100.22	27.12
R. yunnanensis	100.29	27.12
R. yunnanensis	100.22	27.13
R. yunnanensis	101.30	27.13
R. yunnanensis	100.95	27.14
R. yunnanensis	100.22	27.16
R. yunnanensis	100.26	27.18
R. yunnanensis	99.36	27.21
R. yunnanensis	88.87	27.48
R. yunnanensis	99.70	27.48
R. yunnanensis	102.41	27.58
R. yunnanensis	91.90	27.59
R. yunnanensis	99.79	27.61
R. yunnanensis	101.60	27.69
R. yunnanensis	103.20	27.78
R. yunnanensis	102.77	27.80
R. yunnanensis	99.99	27.83
R. yunnanensis	108.68	27.88
R. yunnanensis	99.63	27.91
R. yunnanensis	99.95	27.91
R. yunnanensis	101.62	27.91
R. yunnanensis	108.69	27.92
R. yunnanensis	91.84	27.93
R. yunnanensis	101.29	27.94
R. yunnanensis	99.59	27.97
R. yunnanensis	101.29	27.98
R. yunnanensis	89.39	28.02
R. yunnanensis	99.72	28.02
R. yunnanensis	85.99	28.09
R. yunnanensis	99.87	28.13
R. yunnanensis	100.81	28.16

R. yunnanensis	101.18	28.22
R. yunnanensis	99.36	28.24
R. yunnanensis	98.49	28.27
R. yunnanensis	99.16	28.28
R. yunnanensis	103.56	28.30
R. yunnanensis	98.79	28.36
R. yunnanensis	98.80	28.39
R. yunnanensis	103.72	28.40
R. yunnanensis	103.25	28.41
R. yunnanensis	103.35	28.41
R. yunnanensis	98.96	28.42
R. yunnanensis	98.88	28.53
R. yunnanensis	98.78	28.56
R. yunnanensis	102.53	28.59
R. yunnanensis	101.21	28.60
R. yunnanensis	102.57	28.61
R. yunnanensis	99.82	28.62
R. yunnanensis	99.20	28.67
R. yunnanensis	94.00	28.75
R. yunnanensis	100.96	28.76
R. yunnanensis	102.50	28.85
R. yunnanensis	102.17	28.87
R. yunnanensis	102.12	28.90
R. yunnanensis	102.10	28.94
R. yunnanensis	102.10	28.96
R. yunnanensis	99.91	29.11
R. yunnanensis	101.54	29.21
R. yunnanensis	102.27	29.21
R. yunnanensis	101.52	29.22
R. yunnanensis	103.29	29.23
R. yunnanensis	103.31	29.46
R. yunnanensis	103.31	29.51
R. yunnanensis	103.33	29.55
R. yunnanensis	103.28	29.57
R. yunnanensis	103.35	29.57
R. yunnanensis	102.10	29.58
R. yunnanensis	102.83	29.59
R. yunnanensis	102.66	29.61
R. yunnanensis	102.66	29.67
R. yunnanensis	102.54	29.72
R. yunnanensis	102.24	29.86
R. yunnanensis	102.27	29.86
R. yunnanensis	95.61	29.92
R. yunnanensis	94.78	29.96
R. yunnanensis	100.94	29.99
R. yunnanensis	102.27	29.99
R. yunnanensis	100.93	30.00
R. yunnanensis	101.88	30.00
R. yunnanensis	101.98	30.00

R. yunnanensis	101.87	30.01
R. yunnanensis	101.96	30.04
R. yunnanensis	101.31	30.05
R. yunnanensis	101.82	30.05
R. yunnanensis	101.94	30.13
R. yunnanensis	101.82	30.15
R. yunnanensis	101.99	30.34
R. yunnanensis	102.79	30.37
R. yunnanensis	101.77	30.55
R. yunnanensis	102.12	30.59
R. yunnanensis	102.76	30.70
R. yunnanensis	102.17	30.76
R. yunnanensis	101.90	30.96
R. yunnanensis	102.35	30.98
R. yunnanensis	101.88	31.04
R. yunnanensis	103.17	31.04
R. yunnanensis	102.23	31.05
R. yunnanensis	109.93	31.37
R. yunnanensis	110.03	31.41
R. yunnanensis	103.54	31.50
R. yunnanensis	102.81	31.66
R. yunnanensis	103.80	31.67
R. yunnanensis	101.19	31.81
R. yunnanensis	102.26	31.88
R. yunnanensis	102.20	31.90
R. yunnanensis	101.05	31.95
R. yunnanensis	103.45	31.98

Supplementary Material2 Phtyon script

```
#Reclassify according to the threshold:
import arcpy
from arcpy import env
from arcpy.sa import *
# Check out the ArcGIS Spatial Analyst extension license
arcpy.CheckOutExtension("Spatial")
# Input path
InRasPath = "D:\\OriginalToTif\\"
env.workspace = InRasPath
# Output path
OutRaspath = "D:\\Reclassify\\"
arcpy.env.workspace = InRasPath
files = arcpy.ListFiles("*.tif")
print files
for file in files:
    input = arcpy.Raster(file)
    output = Con(input >0.286516667, 1, 0)
    output.save("D:\\Reclassify\\"+ file)
print "Good job!!!!!"

#Convert to rectangular projection:
import arcpy
from arcpy import env
from arcpy.sa import *
# Check out the ArcGIS Spatial Analyst extension license
arcpy.CheckOutExtension("Spatial")
# Input path
InRasPath = "E:\\raster\\"
env.workspace = InRasPath
# Output path
OutRaspath = "E:\\sin\\"
arcpy.env.workspace = InRasPath
files = arcpy.ListFiles("*.tif")
print files
for file in files:
    arcpy.ProjectRaster_management(file, OutRaspath + "sin_" + file,\
    "PROJCS['SINUSOIDAL_Unspecified_Datum_Semi_major_axis_6371007_181000_Semi_minor_axis_0_0',\
    GEOGCS['',DATUM['D_unknown',SPHEROID['Unknown',6371007.181,0.0]],PRIMEM['Greenwich',0.0],\
    UNIT['Degree',0.0174532925199433]],PROJECTION['Sinusoidal'],PARAMETER['false_easting',0.0],\
    PARAMETER['false_northing',0.0],PARAMETER['central_meridian',0.0],UNIT['Meter',1.0]]",\
    "NEAREST", "1000", "WGS_1984_Major_Auxiliary_Sphere_To_WGS_1984", "", "GEOGCS['GCS_WGS_1984',\
    DATUM['D_WGS_1984',SPHEROID['WGS_1984',6378137.0,298.257223563]],PRIMEM['Greenwich',0.0],\
    UNIT['Degree',0.0174532925199433]]")
    print file + " done"
print "Good job!!!!!"

#Calculate for suitable area:
import xlwt
```

```
import arcpy
arcpy.CheckOutExtension("spatial")
arcpy.env.overwriteOutput = True
inpath = "D:\\int\\"
arcpy.env.workspace = inpath
files = arcpy.ListFiles("*.tif")
outpath = "D:\\Cal_area\\"
wb = xlwt.Workbook()
ws = wb.add_sheet('area')
times = 1
ws.write(0, 0, "IMAGE")
ws.write(0, 1, "AREA")
for file in files:
    print file
    prows = arcpy.UpdateCursor(file)
    ws.write(times, 0, file)
    for prow in prows:
        print prow.VALUE
        if prow.VALUE == 1:
            ws.write(times, 1, prow.COUNT)
        else:
            print "nothing"
    times = times + 1
wb.save(outpath +"cal_area.xls")
print "Good job!!!!!"

#Calculate for mean elevation:
import xlwt
import arcpy
arcpy.CheckOutExtension("spatial")
arcpy.env.overwriteOutput = True
inpath = "D:\\int\\"
arcpy.env.workspace = inpath
files = arcpy.ListFiles("*.tif")
outpath = "D:\\Cal_mean_alt\\"
wb = xlwt.Workbook()
ws = wb.add_sheet('alt')
times = 1
ws.write(0, 0, "IMAGE")
ws.write(0, 1, "Mean Elevation")
elevation = "D:\\alt\\alt.tif"
for file in files:
    ws.write(times, 0, file)
    zs_table = inpath + "output_" + str(times)
    arcpy.gp.ZonalStatisticsAsTable_sa(file, "Value", elevation, zs_table, "DATA", "ALL")
    query_in_row = "Rowid"
    srows = arcpy.SearchCursor (zs_table, "")
    for srow in srows:
        mean = srow.MEAN
```

```
            if srow.VALUE == 1:
                ws.write(times, 1, round(mean))
            else:
                print "nothing"
        del zs_table
        times = times + 1
wb.save(outpath +"mean_alt.xls")
print "Good job!!!!!"

#Calculate for mean longitude and latitude:
import xlwt
import arcpy
arcpy.CheckOutExtension("spatial")
arcpy.env.overwriteOutput = True
inpath = "D:\\int\\"
arcpy.env.workspace = inpath
files = arcpy.ListFiles("*.tif")
outpath = "D:\\mean_lat_lon\\"
wb = xlwt.Workbook()
ws = wb.add_sheet('center')
times = 1
ws.write(0, 0, "IMAGE")
ws.write(0, 1, "Mean")
center = "D:\\XY\\y_cor.tif"
for file in files:
    ws.write(times, 0, file)
    zs_table = inpath + "output_" + str(times)
    arcpy.gp.ZonalStatisticsAsTable_sa(file, "Value", center, zs_table, "DATA", "ALL")
    query_in_row = "Rowid"
    srows = arcpy.SearchCursor (zs_table, "")
    for srow in srows:
        mean = srow.MEAN
        if srow.VALUE == 1:
            ws.write(times, 1, mean)
        else:
            print "nothing"
    del zs_table
    times = times + 1
wb.save(outpath +"mean_center.xls")
print "Good job!!!!!"
```

References

1 Zhang, J., Meng, S., Allen, G. A., Wen, J. & Rao, G. Rapid radiation and dispersal out of the Qinghai-Tibetan Plateau of an alpine plant lineage *Rhodiola* (Crassulaceae). *Molecular Phylogenetics and Evolution* **77**, 147-158 (2014).

2 Gao, Q. *et al.* Chloroplast DNA phylogeography of *Rhodiola alsia* (Crassulaceae) in the Qinghai-Tibet Plateau. *Botany-Botanique* **87**, 1077-1088 (2009).

3 Gao, Q. *et al.* Intraspecific divergences of *Rhodiola alsia* (Crassulaceae) based on plastid DNA and internal transcribed spacer fragments. *Botanical journal of the Linnean Society* **168**, 204-215 (2012).

4 Hou, Y. & Lou, A. Population Genetic Diversity and Structure of a Naturally Isolated Plant Species, Rhodiola

dumulosa (Crassulaceae). *PLoS ONE* **6**, e244979 (2011).

5 Hou, Y. & Lou, A. Phylogeographical patterns of an alpine plant, *Rhodiola dumulosa* (Crassulaceae), inferred from chloroplast DNA sequences. *Journal of Heredity* **105**, 101-110 (2014).

6 Lei, Y. *Genetic diversity and phytochemical diversity of Rhodiola crenulata in China revealed by ISSR markers and essential oils.* Doctor thesis, Fudan University, (2004).

7 Mu, Y., Zhang, Y. & Lou, A. A preliminary study on floral syndrome and breeding system of the rare plant *Rhodiola dumulosa*. *Journal of Plant Ecology* **31**, 528-535 (2007).

8 Xia, T. *et al.* ISSR analysis of genetic diversity of the Qinghai-Tibet Plateau endemic, *Rhodiola chrysanthemifolia* (Crassulaceae). *Biochemical Systematics and Ecology* **35**, 209-214 (2007).

9 Zhang, J., Meng, S. & Rao, G. Phylogeography of *Rhodiola kirilowii* (Crassulaceae): A Story of Miocene Divergence and Quaternary Expansion. *PLoS ONE* **9**, e112923 (2014).

10 Zheng, W. *The research on the ecological character of Longwhip Rhodiola's population on Mt. Sergyela, Tibet.* Doctor thesis, Central South University of Forestry and Technology, (2010).

11 Zhu, L. & Lou, A. Mating system and pollination biology of a high-mountain perennial plant, *Rhodiola dumulosa* (Crassulaceae). *Journal of Plant Ecology* **3**, 219-227 (2010).
